# How traditional cultural load affects tourists’ purchasing intention of tourist souvenirs

**DOI:** 10.1371/journal.pone.0313905

**Published:** 2025-01-09

**Authors:** Qingxi Huang, Dajun Yang, Yixin Xie, Fuqiang Tan, Tingyue Kuang

**Affiliations:** 1 College of History, Culture and Tourism, Gannan Normal University, Ganzhou City, Jiangxi Province, China; 2 School of Administration, North Sichuan Medical College, NanChong City, SiChuan Province, P.R. China; 3 Research Center for Industry Digitalization, Huainan Normal University, Huainan City, AnHui Province, P.R. China; 4 Faculty of Business, City University of Macau, Macao, China; Sichuan Agricultural University, CHINA

## Abstract

Based on the previous studies on the impact of traditional culture on tourists’ purchasing intentions, this study aims to further explore the mechanism and boundary conditions regarding the traditional cultural load in tourist souvenir packaging. Through seven simulated experiments (N = 3203), the impact of different degrees of traditional cultural load on tourists’ purchasing intentions has been examined, with value perception, cultural identity, and purchase purpose, advancing the research in the field of traditional culture and tourism marketing. The findings provide insights for managers in the industry of tourism and souvenir marketing for their package design.

## 1. Introduction

Tourist souvenirs are goods purchased during or after a trip [1]. These goods often have local features and serve as mementos or gifts [2]. Factors such as individuals’ perception of travel importance, attractiveness of the destination, and uniqueness of the souvenirs affect their purchasing intentions [3–5]. To boost sales, merchants integrate local features with traditional culture to increase purchasing intention [6]. The integration of traditional cultural elements has become an effective strategy [7]. Understanding the impact of traditional cultural load in tourist souvenirs is important in developing tourists’ purchasing intention. Traditional cultural load refers to the integration and connotation of traditional culture in a certain product [8]. It stimulates tourists’ consumer behavior by affecting their cultural perceptions [9, 10]. Incorporated traditional culture strengthens tourists’ emotional attachment and brand-consumer connection [11, 12]. However, studies on traditional culture in tourism are limited [13–15]. The impact of traditional cultural load on tourist souvenirs and purchasing intention has been insufficiently explored.

It is certain that there is an extensive body of literature on the topic of consumer purchase intentions for souvenirs, which includes elements within souvenirs (such as traditional patterns, symbols, and craftsmanship) that can represent the cultural characteristics of the destination [16, 17]. However, these studies have largely been conducted from the perspective of how souvenirs convey information to consumers. For instance, tourists tend to purchase souvenirs that reflect the cultural characteristics and craftsmanship level of the destination [18]. Moreover, the design and cultural connotations of souvenirs significantly influence tourists’ purchasing decisions, which includes the assessment of the cultural value of the souvenirs and the enhancement of the sense of cultural identity with the destination [19]. Yet, regrettably, our understanding of the potential impact of the cultural load in souvenirs on tourists’ purchase intentions remains limited. Current research has found that the symbolic meaning of traditional culture in souvenirs subsequently influences tourists’ psychological processes and purchase intentions. For example, when consumers are informed that a product is "handmade," they show greater interest and purchase intention compared to when they are told it is "machine-made" [20]. This indicates that the presence of traditional cultural elements in products is already influencing consumers’ purchasing behavior, making it necessary to delve deeper into this phenomenon. This study is dedicated to addressing the following questions: (1) Does the cultural load of traditional elements in souvenirs (high vs. low) influence tourists’ purchase intentions? (2) What is the mechanism underlying this influence?

To address these gaps, this study proposed a framework to explore the mechanisms and boundary conditions of traditional cultural load on purchasing intentions. It has been argued that different traditional cultural load in tourist souvenirs affect consumer behavior, by influencing perceived value and product type [21–23]. Cultural load affects purchasing intentions through better cultural experiences [24–26]. The framework also considered the mediating role of perceived value and cultural identity, as well as the moderating role of purchasing purpose. By filling these gaps, the study contributed to the understanding of how traditional cultural load in tourist souvenirs influences purchasing behavior.

This study provided valuable insights by examining the impacts of different traditional cultural load on purchasing intentions. It introduced the concept of traditional cultural load and explored its impact on purchasing intentions, expanding the existing literature. This study demonstrated the moderating role of product type and the mediating role of perceived value. It also examined the interaction between cultural identity, cultural load, and purchasing intentions. This study contributed to the understanding of multicultural elements on consumer behavior. Furthermore, the relationship between purchase purpose and consumer behavior has been validated in this study. Practically, the findings guide retailers to increase sales and promote tourist destinations, by selling souvenirs with higher traditional cultural load.

## 2 Literature review and hypotheses

### 2.1 Traditional cultural load and tourists’ consumer behavior

Based on previous research findings, this study proposed the concept of traditional cultural load, which refers to the positive impact of products combining with traditional cultural elements on consumers’ purchasing intentions in a modern business environment [27, 28]. First, the traditional cultural load in products can evoke emotional resonance [29]. Traditional culture represents the unique history and cultural heritage of a nation or a region, and it carries strong emotional value. Traditional culture-enabled products could evoke consumers’ emotional resonance, because of their affection and identification with traditional culture, which can stimulate their purchasing intentions of related products. Second, the traditional cultural load in products is a symbol of cultural confidence [30]. As cultural diversity has been valued, people are more and more aware of the need to identify and protect the traditional culture. Purchasing traditional culture-enabled products is not only to meet the demand for goods, but also a demonstration of their own cultural identity. This kind of purchasing motivation enhances consumers’ purchasing intentions and makes them more inclined to choose traditional culture-enabled products. Last, the traditional cultural load in product can meet the consumers’ needs for personalization [31]. To better integrate traditional culture into products, manufacturers pay more attention to the differentiation and personalization of products. The flexible application of traditional cultural elements enables products to meet the needs of different consumers and better fit their pursuit of personalized products. Therefore, the advantages of traditional cultural load in products lie in their capability of enhancing consumers’ purchasing intentions, by fulfilling their personalized needs.

The traditional cultural load in tourist souvenirs can positively contribute to several aspects. On the one hand, it increases the differentiation of tourism products [32]. Traditional culture-enabled tourism products provide tourists with a distinctive tourism experience by incorporating unique traditional cultural elements. Such differentiated features and uniqueness of product increase tourists’ purchasing intentions by attracting their attention. On the other hand, it enhances tourists’ cultural awareness of tourism products [33]. With the help of rich cultural connotations and experiences, traditional culture-enabled tourism products provide educational opportunities, helping tourists to gain a deeper understanding of the traditional culture of the destination [34]. Tourists’ need to broaden their horizons and enhance their cultural awareness also motivates them to purchase related tourism products, which shapes the word-of-mouth and brand image of tourism products [35]. Traditional culture-enabled tourism products usually provide tourists a unique and valuable experience, making sure there is a high level of satisfaction. Through positive word-of-mouth communication and positive comments, companies are able to build a favorable brand image, and attract more tourists to experience, so as to increase their purchasing intentions.

Based on above analysis, the following hypothesis is proposed.

H1: Compared those with low traditional cultural load, tourist souvenirs with high traditional cultural load are more likely to stimulate tourists’ consumer behavior.

### 2.2 Cultural identity and purchasing intentions

The cultural identity theory was proposed by the famous American psychoanalyst Erikson in the early 1950s [36]. It confirmed the spiritual value of human beings, which is mainly inherited through the national identity, traditional customs and lifestyles, as well as through the "collective unconscious" [37]. It emphasizes the affirmative recognition of an ethnic community, which they have developed over a long period of time by living together [38]. The integration of people’s various identities reduces cultural conflicts caused by deviations or heterogeneities [39]. Researches have shown that, on the one hand, cultural identity affects consumers’ purchase decision-making [40]. For example, consumers’ cultural identity may affect their purchase motivation, by leading them to choose certain products that are in line with their cultural identity or social status [41]. On the other hand, cultural identity affects consumer loyalty [42]. For instance, consumers are more committed to purchase product from brands that represent their cultural identity or are aligned with their cultural values and images [43]. Previous studies have proved that cultural identity is one of the important factors that affect consumer behavior in several ways [44].

Cultural identity theory can be widely applied in tourism consumption as well as in real life. Since high cultural identity can help to build tourists’ place attachment and their cultural connectivity with the travel destinations, which contributes to the cultural bond between them [45]. In addition, there is some empirical evidence suggested that cultural identity increases tourists’ inclination to spend money in travel destinations, and affect tourists’ perceptions of the sustainable development within travel destinations [46]. The study conducted by Zou et al. (2022) further suggests that cultural identity contributes to the cultural symbols standing for the travel destination, thereby tourists could learn better about it. Cultural identity could also be taken as a cognitive image in tourism marketing [47], which plays an important role in tourists’ image perception of travel destination and boosts the value co-creation of tourism [48]. According to cultural identity theory, Zhang et al. (2019) verified that cultural identity moderates tourists’ mind-flow experience of tourism activities, which affects tourists’ perceived value of tourism products. Therefore, this study adopted cultural identity theory to explore the impact of traditional cultural load in tourist souvenirs on tourists’ potential purchasing intentions.

Based on above analysis, the following hypothesis is proposed.

H2: Cultural identity (high vs. low) moderates the relationship between traditional cultural load (high vs. low) in tourist souvenirs and tourists’ purchasing intentions.

### 2.3 The moderating role of product type

Product type is one of the important antecedent factors that affect consumers’ purchasing intention [49]. Products can be classified into different categories or types, based on their differences in characteristics, functions, and uses [50]. The concept of product type helps to classify different products and provides an organizational structur based on common attributes, which is important in market research, product development, market positioning and marketing strategy-making [51]. Previous studies have demonstrated that product types can be categorized into "utilitarian" and "hedonic". Utilitarian products emphasize the function of the product, whereas hedonic products emphasize the pleasant experience and self-expression that the product provides to the consumers [52]. And different product types have different characteristics and functions that can attract different types of consumers [53].

Previous findings indicated that product type moderates purchasing intentions, particularly in the dimensions of utilitarian and hedonic products [54]. Utilitarian products are goods that fulfill people’s material needs, as well as serve great practical functions and benefits. These products are usually closely related to personal and family life, such as home appliances, automobiles, and daily necessities. Customers purchase utilitarian products mainly to meet their living needs, as well as to reach efficiency and convenience [55]. Their purchasing intentions are usually involved with comprehensive comparative analysis, in order to make sure the performance, quality and function of the product can satisfy the actual needs, which would be greatly influenced by practicality and reliability. Hedonic products refer to goods that can meet people’s spiritual needs, as well as bring fun and pleasure [56]. These products are usually associated with entertainment and leisure activities, such as traveling, video games, and music. Customers purchase hedonic products mainly to pursue and enjoy the emotional fulfillment with great pleasure and delight [57]. Their purchasing intentions are usually related to the entertainment, excitement, and emotional value, and the impact of products on personal psychological well-being is always needed to be taken into consideration [52], which means their purchasing intentions are easily affected by emotional factors.

Based on above analysis, the following hypotheses are proposed.

H3: Utilitarian value (high vs. low) moderates the relationship between traditional cultural load (high vs. low) in tourist souvenirs and tourists’ purchasing intentions.
H4: Hedonic value (high VS. low) moderates the relationship between traditional cultural load (high VS. low) in tourist souvenirs and tourists’ purchasing intentions.

### 2.4 Perceived value and purchasing intentions

In 1988, Zaithamal first proposed the theory of customer perceived value from the perspective of the customer [58]. Perceived value refers to the overall evaluation of the utility of products or services, after balancing the benefits that customers can perceive against the costs they have to pay. Zaithamal argued that customer orientation should be adopted when companies are designing, creating, and delivering value to their customers, which means customers’ perception of value should be taken as a determining factor. Customer value is determined by customers rather than any company, which is actually Customer Perceived Value (CPV) [59].

Perceived social value refers to the extent to which individuals believe that a product, service, or behavior has a positive impact on the society [60]. Individuals tend to perceive a product or service with high social value when they believe that this product or service can improve people’s quality of life, release poverty, or provide fair opportunities. Perceived social value could be more significant for some programs about environmental products, public welfare activities, and social responsibility [61]. It can affect purchasing intentions in multiple ways. On the one hand, individuals are more inclined to purchase products or services, since they believe that their consumption could meet their needs and contribute to society at the same time [62]. On the other hand, perceived social value can also enhance individuals’ moral mentality, so they may pay higher prices for products or services with higher social value.

Perceived heritage value refers to individuals believe a product, service, or behavior is important for the inheritance and preservation of culture, traditions, or values [63]. Individuals tend to perceive a product or service with high heritage value, when they believe that this product or service can inherit and protect their cherished cultural heritage, traditional custom, or values [64]. The perceived heritage value could be more significant with traditional handicrafts, cultural activities or educational programs. It can affect purchasing intentions in multiple ways. On the one hand, individuals may purchase products or services with heritage value out of their need for cultural identity, self-identity or cultural inheritance. This kind of consumer behavior could be taken as the affirmation and expression of their cultural identity. On the other hand, perceived heritage value could evoke emotional resonance and social needs among individuals, so they are willing to purchase products or services with heritage value [65].

From the perspective of perceived social value of tourist souvenirs, consumers can perceive the social significance and social identity that embodied in tourist souvenirs. Tourist souvenirs usually reflect the characteristics of the travel destination in culture, history, and geography, and customers’ purchase allows them to establish a connection and emotional resonance with it [66]. Consumers may perceive these souvenirs with high perceived social value if they consider their purchase as a way to support and approve the local society and culture, thus enhancing purchasing intentions. From the perspective of perceived heritage value of tourist souvenirs, consumers can perceive the value of the history and its inherence that the tourist souvenirs represent [67]. Some tourist souvenirs may possess a unique historical or cultural background, standing for a tradition or far-reaching commemorative significance. Consumers may perceive these souvenirs with high perceived heritage and consider their consumption as a way to inherit and commemorate, thus increasing purchasing intentions.

In summary, consumers’ purchasing intentions for tourist souvenirs is affected by perceived social value and perceived heritage value. If consumers value the social significance of a travel destination, their purchasing intentions will be enhanced by souvenirs that reflect local social values and identity. Consumers’ purchasing intentions for souvenirs with unique historical or cultural backgrounds will be increased, if they are interested in the represented historical and heritage values.

Based on above analysis, the following hypotheses are proposed.

H5: Perceived social value mediates the relationship between traditional cultural load (high vs. low) in tourist souvenirs and tourists’ purchasing intentions.
H6: Perceived heritage value mediates the relationship between traditional cultural load (high vs. low) in tourist souvenirs and tourists’ purchasing intentions.

### 2.5 Moderating role of purchase purpose (for oneself vs. for others)

People’s purchasing is not only for their own, but also often for others, and these difference in purchasing purposes directly affect consumers’ purchasing decision-making [68]. It has been found that when consumers purchase for themselves and personally own the product, they are inclined to view the brand as an extension of themselves and to project the brands’ characteristics onto themselves [69]. Individuals with a strong competitiveness always prefer competency-based brands, in the hope of conveying to others that they are highly competent as well. However, when consumers purchase for others, the product does not belong to themselves, which will no longer be taken as an extension of themselves [70]. It means that different purchasing purposes (for oneself vs. for others) can significantly affect consumers’ purchasing intentions.

In purchasing tourist souvenirs, the tourists’ purchase purpose may involve differences between themselves and others. From the perspective of purchasing for oneself, tourists’ purchase purpose could be such as memorizing the trip for themselves, showing their interest in a particular area, or collecting specific souvenirs [71]. In this case, tourists’ purchasing intentions are mainly driven by personal preferences and interests, which are less related to traditional cultural load. From the perspective of purchasing for others, tourists may purchase tourist souvenirs be given to people, such as family, friends or other collectors. In this case, tourists’ purchasing intentions may be more affected by others’ preferences and identification with traditional culture. Tourists may be inclined to purchase traditional culture-enabled products related to the target receiver to show their concern and respect.

Based on above analysis, the following hypothesis is proposed.

H7: Purchasing purpose (for oneself vs. for others) moderates the relationship between traditional cultural load (high vs. low) in tourist souvenirs and tourists’ purchasing intentions.

**Fig 1:**
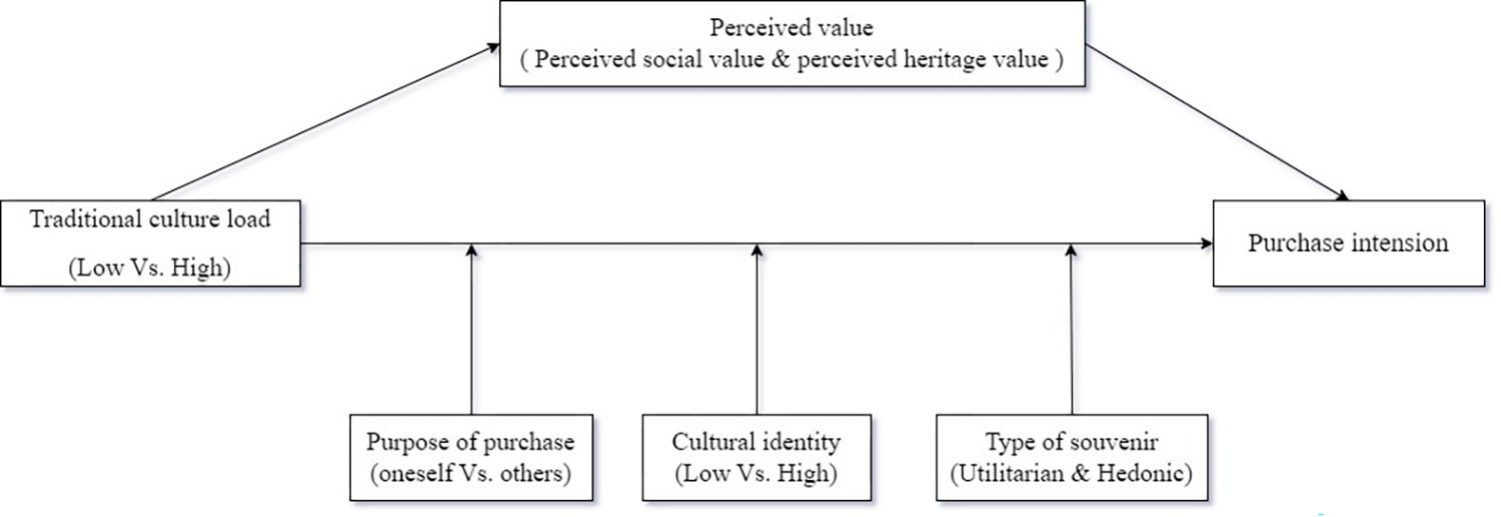
Conceptual model diagram.

The conceptual model is shown in Fig 1.

## 3. Overview of studies

This study conducted the following seven related researches to verify the above seven hypotheses. Experiment 1 validated that traditional cultural load can increase tourists’ purchasing intentions of tourist souvenirs (H1). Experiment 2 validated the moderating role of product type (utilitarian products) among the impact of traditional cultural load in tourist souvenirs on tourists’ purchasing intentions (H2). Experiment 3 validated the moderating role of product type (hedonic products) among the impact of traditional cultural load in tourist souvenirs on tourists’ purchasing intentions (H3). Experiment 4 analyzed the mediating role of product perceived value (perceived social value) among the impact of traditional cultural load in tourist souvenirs on tourists’ purchasing intentions (H4). Experiment 5 analyzed the mediating role of product perceived value (perceived heritage value) among the impact of traditional cultural load in tourist souvenirs on tourists’ purchasing intentions (H5). Experiment 6 analyzed the moderating role of cultural identity among the impact of traditional cultural load in tourist souvenirs on tourists’ purchasing intentions (H6). Experiment 7 examined the moderating role of purchase purpose (for self vs. for others) among the impact of traditional cultural load in tourist souvenirs on tourists’ purchasing intentions (H7). Our experimental data collection began in December 2023 and ended in February 2024 (See Table 1 for details). All questionnaire contents are shown in Table 2.

**Table 1:** Relevant experimental design of 7 experiments.

Study	Study1	Study2	Study3	Study4	Study5	Study6	Study7
Purpose	To test for main effects (H1)	To test the modulation effect of practical product type (H2)	To test the modulation effect of hedonic product type (H3)	To test the mediating effect of perceived social value(H4)	To test the mediating effect of perceived inherited value(H5)	To test the modulation effect of cultural identity (H6)	To test the modulation effect of purpose of purchase (H7)
Independent Variable	Traditional Cultural Load (High Vs. Low)	Traditional Cultural Load (High Vs. Low)	Traditional Cultural Load (High Vs. Low)	Traditional Cultural Load (High Vs. Low)	Traditional Cultural Load (High Vs. Low)	Traditional Cultural Load (High Vs. Low)	Traditional Cultural Load (High Vs. Low)
Dependent variable	Purchase intention of tourist souvenirs	Purchase intention of tourist souvenirs	Purchase intention of tourist souvenirs	Purchase intention of tourist souvenirs	Purchase intention of tourist souvenirs	Purchase intention of tourist souvenirs	Purchase intention of tourist souvenirs
Mediators	-	-	-	Perceived social value	Perceived inherited value	-	-
Moderator	-	Practical product type	Hedonic product type	-	-	Cultural identity	Purpose of purchase
Methods	ANOVA	ANOVA PROCESS 1	ANOVA PROCESS 1	ANOVA PROCESS 4	ANOVA PROCESS 4	ANOVA PROCESS 1	ANOVA PROCESS 1
Results	Supported H1	Supported H2	Supported H3	Supported H4	Supported H5	Supported H6	Supported H7

**Table 2:** Measurement variables and their measurement problems.

Variable	Measurement problem	Scale source
**Gender**	Male	
	Female	
**Age**	18–25 years old	
	26–40 years old	
	41–60 years old	
	Over 61 years old	
**Education**	Primary school	
**background**		
	Junior high school	
	Technical secondary school,	
	high school	
	Undergraduate college	
	Postgraduate	
	Doctor-postgraduate	
**Traditional cultural load**	How much traditional culture do you think the product contains?	
**Utilitarian**	Do you agree that the above-mentioned tourist souvenirs with cultural loads meet your functional needs for such products?	[73]
**Hedonic**	Do you agree that the above tourist souvenirs with cultural loads can bring you pleasure and make you feel happy during the shopping process?	[75]
**Perceived social value**	Do you agree that the above tourist souvenirs with cultural connotations help you to be recognized by others?	[76]
	Do you agree that this product will leave a good impression on others?	
	Do you agree that this product will improve others’ perception of you?	
**Perceived inheritance value**	Do you agree that the purchase of tourist souvenirs with cultural connotations can help to promote the historical heritage of the products?	[77, 78]
	Do you agree that buying this product promotes the brand’s longevity?	
**cultural identity**	Do you agree that you are willing to spend a lot of time to learn about its history, traditions and customs?	[79]
	Do you agree that you have a strong sense of belonging and attachment to your own ethnic group?	
	Do you agree that you often do things that help you better understand your ethnic background?	
**Purpose of Purchase**	Do you agree that you are willing to buy the above souvenirs containing traditional cultural contents for friends or family?	
	Do you agree that you are willing to buy for yourself the traditional cultural payload of the tourist souvenirs?	
**Purchase intension**	Do you agree that when you understand the cultural heritage of the product, you have a great possibility to buy cultural tourism souvenirs in the scenic spot?	[72]

For studies involving human participants and all experimental methods, this study is confirmed to have obtained the approval of the Academic Committee of Gannan Normal University, confirmed that all studies were conducted in accordance with relevant guidelines/regulations. The subjects in this study were recruited from the Internet immediately, and no specific subject consent procedures were required, and the approval and consent of the Academic Committee of Gannan Normal University were obtained.

We conducted longitudinal experimental data analysis for the aforementioned seven experiments, and the longitudinal experimental results once again confirmed our research hypotheses. The purposes of conducting longitudinal experiments are to: (1) observe trends: Longitudinal research allows investigators to monitor changes in participants’ attitudes, behaviors, or cognitions over time. Such variations may be due to experimental interventions, seasonal factors, environmental changes, or other time-related variables. (2) enhance the reliability of the results: Repeated measurements can mitigate the impact of random errors and provide more reliable data. By comparing data from different time points, the experimental effects can be estimated more accurately. (3) strengthen causal inference: Although longitudinal research itself does not establish causal relationships, it can provide clues for causal inference by demonstrating patterns of change over time. If the experimental effects are consistent over time and align with the expected causal relationships, this can reinforce the argument for causality. The specific longitudinal experimental results are presented in Appendix X (S2 File).

## 4 Experiment 1: Traditional cultural load in tourist souvenirs- purchasing intentions

### 4.1 Experimental design

Experiment 1 took the one-way between-subjects design (traditional cultural load: high vs. low) in order to investigate the main effect of traditional cultural load in tourist souvenirs on tourists’ purchasing intentions (H1). There were 405 participants randomly recruited on Credamo, among which 3 questionnaires were excluded due to short answering time and invariant responses (selecting the same answer repeatedly). A valid sample of 402 participants was collected, of which 202 (50.2%) were males and 200 (49.8%) were females. The age distributions of the participants were 47.5% (191) under the age of 18, 2.7% (11) aged between 18 and 25, 42.5% (171) aged between 26 and 40, 4.2% (17) aged between 41 and 60, and 3% (12) aged 61 and above. They were randomly divided into two tourism scenarios of tea culture, one group of 202 participants with low traditional culture loaded products, and the other group of 200 participants with high traditional culture loaded tourist souvenirs.

**Fig 2:**
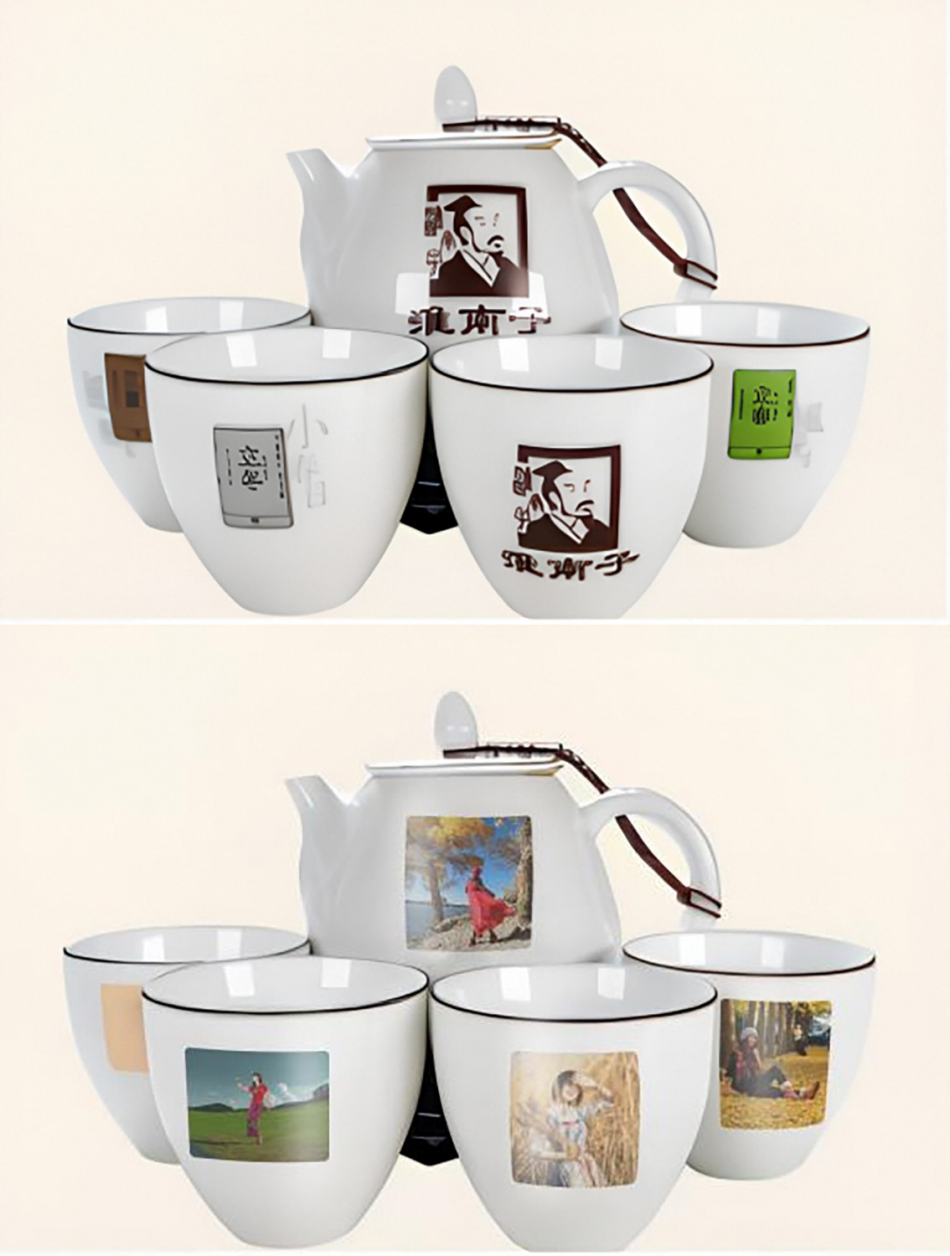
a. High traditional culture loading group stimulus material in experiment 1. b. Low traditional culture loading group stimulus material in experiment 1.

The tea set with the elements of Huainanzi culture was taken as the stimulus material for the group of high cultural load, in a scene of the scenic spot with tea culture in mountainous areas, meanwhile, in the group of low cultural load, an ordinary no patterned tea set was taken as the stimulus material (see Fig 1). Participants were asked to imagine that they were in a large shopping mall in a scenic spot with tea culture and were personally interested in traditional culture, and there were tourist souvenirs on the shelves. Then the participants were led to answer the following questions, "How much do you think the traditional cultural load is in this product?", "Do you agree that there is a great potential for you to purchase a cultural tourist souvenir from scenic spots after you learn about the cultural load in it" [72]. After completing the experiment, researchers collected demographic information of the participants (See Fig 2A and 2B for details).

### 4.2 Experimental result

There was a main effect analysis. The researchers conducted a one-way ANOVA with purchasing intentions of tourist souvenirs as the dependent variable and traditional cultural load as the independent variable. The results showed that the purchasing intentions M _high cultural load_ = 5.83, SD _high cultural load_ = 1.557; M _low cultural load_ = 5.83, SD _low cultural load_ = 1.557, F(1,400) = 320.056, P<0.001. It indicated that the independent variable has a significant impact on the dependent variable, and tourists’ purchasing intentions for tourist souvenirs with high cultural load is significantly higher than those with low cultural load. Therefore, H1 is validated.

### 4.3 Discussion

Experiment 1 verified the main effect of traditional cultural load in tourist souvenirs on tourists’ purchasing intentions in scenic spots, which validated H1. This indicated that traditional cultural load in scenic spots positively predicts tourists’ purchasing intentions of tourist souvenirs. However, it failed to elaborate the internal mechanism and boundary conditions of traditional cultural load in tourist souvenirs affecting tourists’ purchasing intention.

In order to compensate for those shortcomings, Experiment 2 introduced the product type as a moderating variable to analyze the interaction effect of traditional cultural load in tourist souvenirs in scenic spots and tourists’ purchasing intentions, so as to better understand the mechanism by which traditional cultural load in tourist souvenirs in scenic spots affect tourists’ purchasing intentions.

## 5 Experiment 2: Moderating role of product type (Utilitarian)

### 5.1 Experimental design

Experiment 2 took the one-way between-subjects design (traditional cultural load: high vs. low) in order to discuss the moderating role of utilitarian products in the scenic spot on the relationship between the traditional cultural load in tourist souvenirs and tourists’ purchasing intentions, from the perspective of stimulation from product type (utilitarian products)(H2), and to investigate the main effect of traditional cultural load in tourist souvenirs on tourists’ purchasing intentions (H1). There were 399 participants randomly recruited on Credamo, of which 199 (49.9%) were males and 200 (50.1%) were females. The age distribution of the participants was 47.4% (189) under the age of 18, 3.8% (15) aged 18–25, 42.6% (170) aged 26–40, 2.8% (11) aged 41–60, and 3.5% (14) aged 61 and above. They were randomly divided into two tourism scenarios of tea culture, one group of 199 participants with low traditional culture loaded products, and the other group of 200 participants with high traditional culture loaded tourist souvenirs.

**Fig 3:**
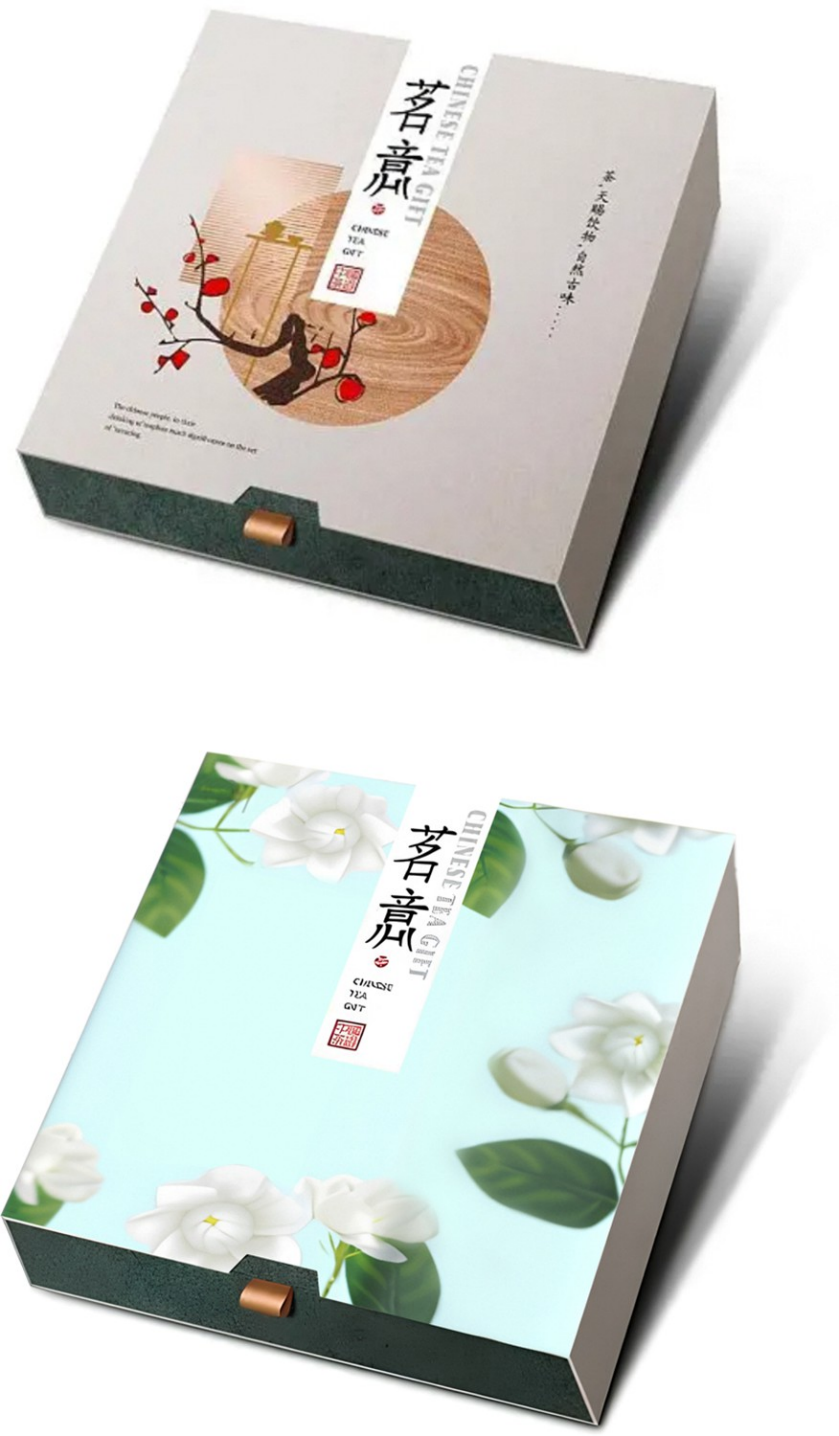
a. High traditional culture loading group stimulus material in experiment 2. b. High traditional culture loading group stimulus material in experiment 2.

Participants were asked to imagine that they were in a large shopping mall in a scenic spot, where they saw a variety of videos introducing tea brewing techniques, books on the inheritance of the tea art, and various tea products in different packages. Then the participants were led to answer the following questions, "How much do you think the traditional cultural load is in this product?", "Do you agree that the tourist souvenirs with cultural load mentioned above fulfill your functional needs for this product type?" (1 = strongly disagree, 7 = strongly agree) [73], and "Do you agree that there is a great potential for you to purchase a cultural tourist souvenir from scenic spots after you learn about the cultural load in it" (1 = strongly disagree, 7 = strongly agree) [72]. After completing the experiment, researchers collected demographic information of the participants (Cronbach’s α = 0.762). In the group of high cultural load, book-style tea box packaging was taken as the stimulus material, in the group of low cultural load, an ordinary can tea packaging was taken as the stimulus material (See Fig 3A and 3B for details).

### 5.2 Experimental result

There was a manipulation check. The utilitarian value of the product was manipulated in Experiment 2. The results showed that M _high utilitarian value_ = 5.99, SD _high utilitarian value_ = 1.06; M _low utilitarian value_ = 4.29, SD _low utilitarian value_ = 1.01, F (1, 397) = 269.534, p < 0.001. Therefore, the manipulations are effective in Experiment 2.

There was a main effect analysis. Experiment 2 conducted an analysis of variance (ANOVA) with purchasing intentions of tourist souvenirs as the dependent variable and traditional cultural load as the independent variable. The results showed that the purchasing intentions of tourist souvenirs in the high cultural load group (M = 5.99, SD = 1.219) was significantly higher than that of the low cultural load group (M = 4.33, SD = 0.857, F (1, 397) = 247.88, p<0.001). H1 was validated.

**Fig 4:**
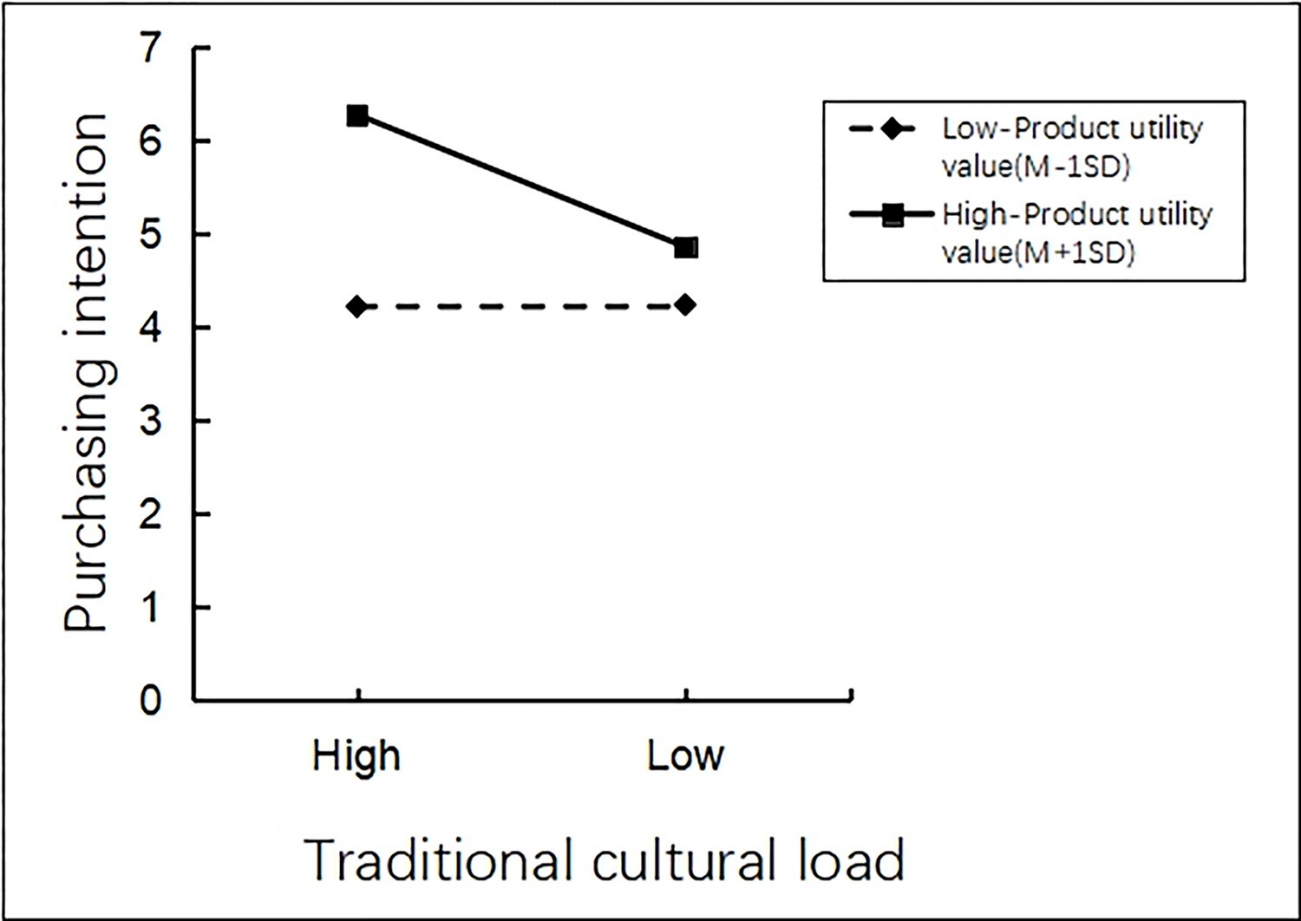
Interaction between utilitarian product type and traditional cultural load.

There was an interaction effect analysis. The researchers took traditional cultural load (high vs. low) as the independent variable, product utilitarian value as the moderating variable, and tourists’ purchasing intentions of tourist souvenirs as the dependent variable. Process model 1 was employed to analyze the interaction effect of product utilitarian value and traditional cultural load on tourists’ purchasing intentions of tourist souvenirs (Bootstrap sample: 5000 [74]). The results showed that the main effect of traditional cultural load on tourists’ purchasing intention of tourist souvenirs in scenic spots was significant (β = -0.7009, P<0.001,95% CI [-0.9642~-0.4377]). The moderating effect of product utilitarian value on tourists’ purchasing intentions of tourist souvenirs is significant (β = 0.3298, P<0.001, 95% CI [0.2646~0.3951]). The interaction effect of product utilitarian value and traditional cultural load on tourists’ purchasing intentions of tourist souvenirs was significant (β = -0.3552, P<0.001,95% CI [-0.4857~-0.2247]). It can be seen that product utilitarian value can effectively moderate the relationship between traditional cultural load and tourists’ purchasing intentions of tourist souvenirs (See Fig 4 for details).

### 5.3 Discussion

Experiment 2 verified that product utilitarian value can effectively moderate the impact of traditional cultural load on tourists’ purchasing intentions of tourist souvenirs. Taking into account the utilitarian attributes of products, tourists are more inclined to purchase tourist souvenirs with higher utilitarian value and higher traditional cultural loads. When product with the same cultural load, tourists were favored the products with higher utilitarian value. However, Experiment 2 only examined the attributes of utilitarian value in the product types and failed to examine the hedonic attributes in the product types.

In order to compensate those shortcomings, Experiment 3 introduced the hedonic element in product type as a moderating variable to analyze the interaction effect between traditional cultural loads in scenic spots and tourists’ purchasing intentions of tourist souvenirs, and attempted to further explore that tourists prefer hedonic element or utilitarian elements in their purchasing.

## 6 Experiment 3: Moderating role of product type (Hedonic)

### 6.1 Experimental design

Experiment 3 conducted a 2 (traditional cultural load: high vs. low) X 2 (product hedonic attributes: high vs. low) ANOVA in order to discuss the moderating role of hedonic products in the scenic spot on the relationship between the traditional cultural load in tourist souvenirs and tourists’ purchasing intentions, from the perspective of stimulation from product type (hedonic products)(H3), and to investigate the main effect of traditional cultural load in tourist souvenirs on tourists’ purchasing intentions (H1). There were 401 participants randomly recruited on Credamo, of which 201 (50.1%) were males and 200 (49.9%) were females. The age distribution of the participants was 4.5% (18) under the age of 18, 47.6% (191) aged between 18–25, 42.4% (170) aged between 26–40, 3.7% (15) aged between 41–60, and 1.7% (7) aged 61 and above. They were randomly divided into two tourism scenarios of tea culture, one group of 200 participants with low traditional culture loaded products, and the other group of 199 participants with high traditional culture loaded tourist souvenirs.

**Fig 5:**
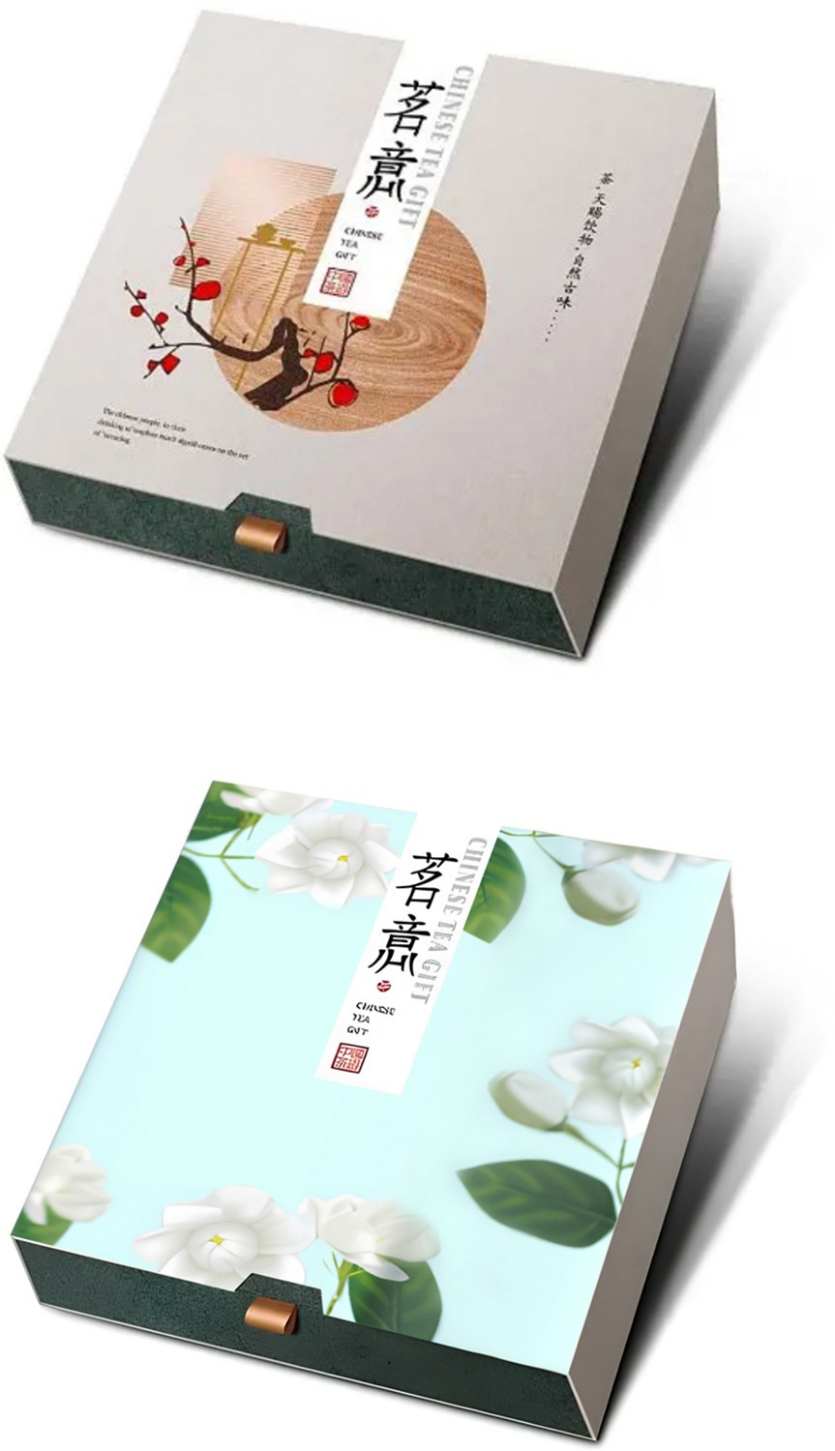
a. High traditional culture loading group stimulus material in experiment 3. b. Low traditional culture loading group stimulus material in experiment 3.

The materials for guidance in Experiment 3 were the same as in Experiment 2. The participants were led to answer the following questions, "How much do you think the traditional cultural load is in this product?", "Do you agree that the tourist souvenirs with cultural loads mentioned above give you a sense of pleasure and make you feel happy in your shopping?" (1 = strongly disagree, 7 = strongly agree) [75], and "Do you agree that there is a great potential for you to purchase a cultural tourist souvenir from scenic spots after you learn about the cultural load in it"(1 = strongly disagree, 7 = strongly agree) [72]. After completing the experiment, researchers collected demographic information of the participants (Cronbach’s α = 0.73). In the group of high cultural load, book-style tea box packaging was taken as the stimulus material, in the group of low cultural load, an ordinary can tea packaging was taken as the stimulus material (See Fig 5A and 5B for details).

### 6.2 Experimental result

There was a manipulation check. Experiment 3 manipulated the product’s hedonic value. The results showed that M _high hedonic value_ = 5.67, SD _high hedonic value_ = 1.413; M _low hedonic value_ = 4.05, SD _low hedonic value_ = 0.986, F (1, 399) = 175.178, p<0.001. Therefore, the manipulations are effective in Experiment 3.

There was a main effect analysis. The researchers conducted an analysis of variance (ANOVA) with tourists’ purchasing intentions of tourist souvenirs as the dependent variable and traditional cultural load as the independent variable. The results showed that M _high cultural load_ = 5.7, SD _high cultural load_ = 1.273, M _low cultural load_ = 4.33, SD _low cultural load_ = 0.857, F (1, 397) = 247.88, p<0.001), indicating that the purchasing intentions of high traditional cultural load group is significantly higher than that of low traditional cultural load group. It is obvious that traditional cultural load in tourist souvenirs has a significant impact on tourists’ purchasing intentions. H1 was verified.

**Fig 6:**
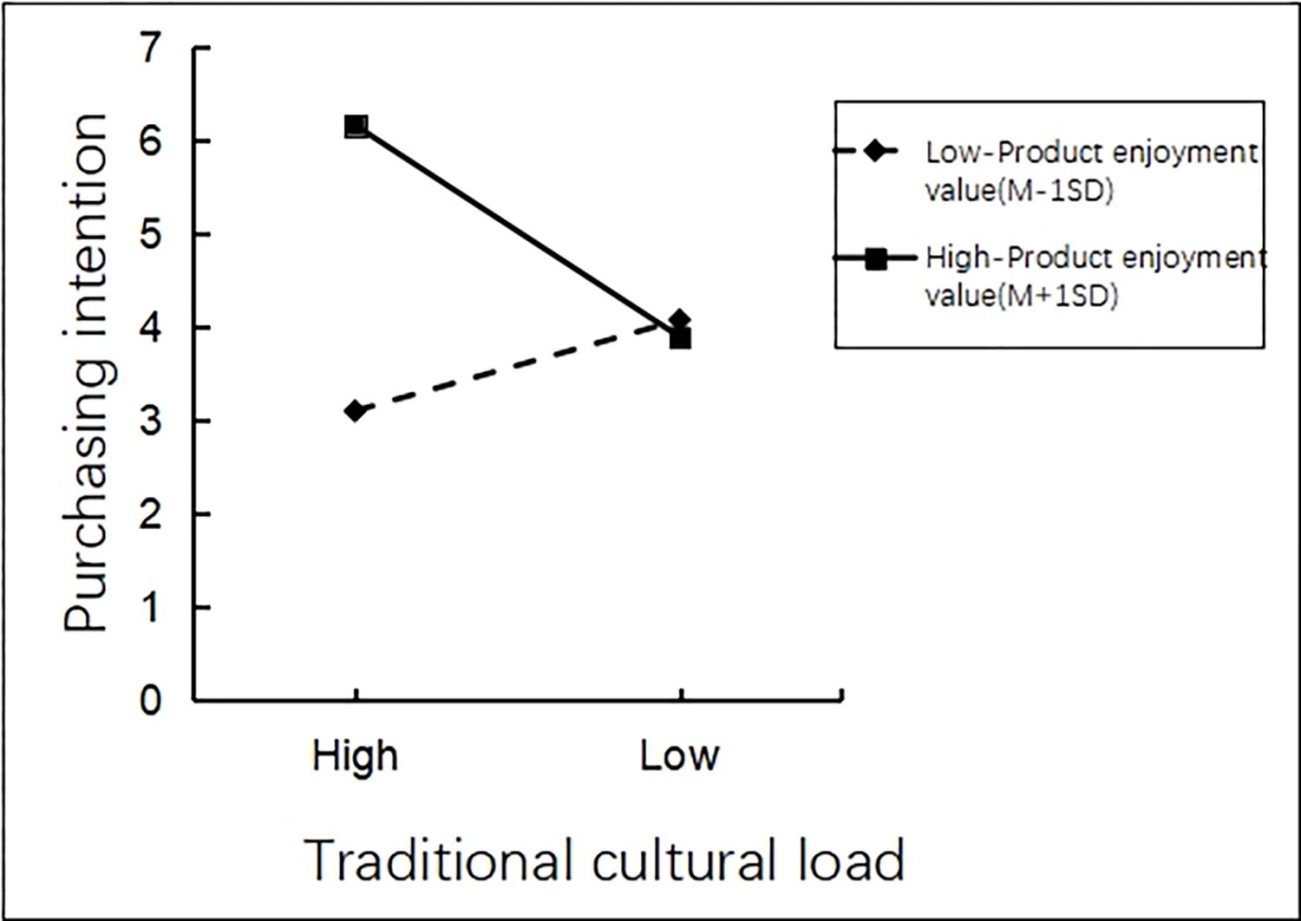
Interaction between hedonic product type and traditional cultural load.

There was an interaction effect analysis. The researchers took traditional cultural load as the independent variable, product hedonic value as the moderating variable, and tourists’ purchasing intention of tourist souvenirs as the dependent variable. Process model 1 was adopted to analyze the moderating effect of hedonic value on the relationship between traditional cultural load and tourists’ purchasing intentions of tourist souvenirs (Bootstrap sample: 5000; [74]). The results showed that the main effect of traditional cultural load on tourists’ purchasing intentions of tourist souvenirs in scenic spots was significant (β = -0.6469, P<0.001, 95% CI [-0.917~-0.3769]). The moderating effect of product hedonic value on the relationship between traditional cultural load and tourists’ purchasing intentions of tourist souvenirs was significant (β = 0.3588, P<0.001, 95% CI [0.2914~0.4263]). The interaction effect of product hedonic value and traditional cultural load on tourists’ purchasing intentions of tourist souvenirs was significant (β = -0.8041, P<0.001, 95% CI [-0.9389~-0.6693]). It can be seen that product hedonic value can effectively moderate the relationship between traditional cultural load and tourists’ purchasing intentions of tourist souvenirs (See Fig 6 for details).

### 6.3 Discussion

Experiment 3 further demonstrated the moderating effect of the hedonic value of product on the relationship between traditional cultural load and tourists’ purchasing intentions of tourist souvenirs. The results showed that tourists prefer to purchase souvenirs with high hedonic value in a high cultural load context, while prefer to purchase souvenirs with low hedonic value in a low cultural load context. However, Experiment 3 failed to take into account the impact of tourists’ perceived value of tourist souvenirs on their purchasing intentions. Tourists’ perceptions of the cultural heritage and social value carried by tourist souvenirs may also affect their purchasing intentions.

In order to compensate those shortcomings, Experiment 4 examined the mediating role of tourists’ perceived social value on the impact of traditional cultural load on tourists’ purchasing intentions of tourist souvenirs, and further analyzed its internal mechanism and boundary conditions.

## 7 Experiment 4: The mediating role of perceived value (perceived social value)

### 7.1 Experimental design

Experiment 4 took the one-way between-subjects design (traditional cultural load: high vs. low) in order to discuss the mediating role of perceived social value on the relationship between the traditional cultural load in tourist souvenirs and tourists’ purchasing intentions (H4), and to investigate the main effect of traditional cultural load in tourist souvenirs on tourists’ purchasing intention (H1). There were 400 participants randomly recruited on Credamo, of which 200 (50%) were males and 200 (50%) were females. The age distribution of the participants was 3.5% (14) under 18 years old, 47.5% (190) aged between 18–25 years old, 42.5% (170) aged between 26–40 years old, 3.5% (14) aged between 41–60 years old, and 3% (12) aged 61 years old and above. They were randomly divided into two scenic spots of traditional tea town, one group of 200 participants with low traditional culture loaded products, and the other group of 200 participants with high traditional culture loaded tourist souvenirs.

**Fig 7:**
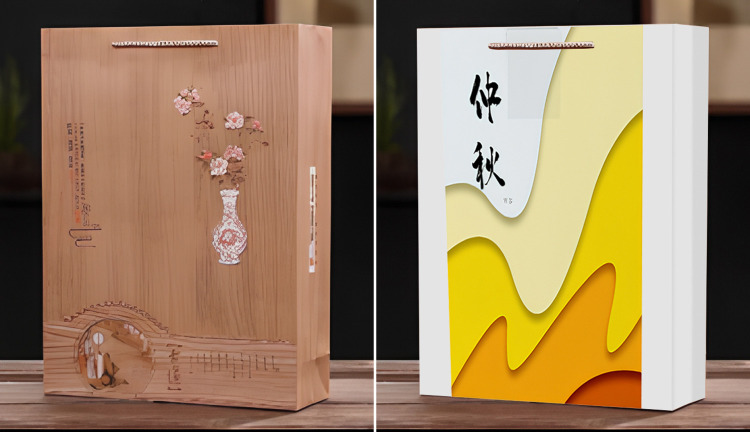
a. High traditional culture loading group stimulus material in experiment 4. b. Low traditional culture loading group stimulus material in experiment 4.

Participants were asked to imagine that they were in a large shopping mall in a scenic spot of traditional tea town, where has many products that mix traditional elements with modern ones. Then participants were led to answer questions, such as "How much do you think the traditional cultural load is in this product?", "Do you agree that the tourist souvenir with cultural load mentioned above will help you to be acknowledged by others", "Do you agree that this product will leave a good impression on other people" (1 = Strongly Disagree, 7 = Strongly Agree) [76], and "Do you agree that there is a great potential for you to purchase a cultural tourist souvenir from scenic spots after you learn about the cultural load in it"(1 = strongly disagree, 7 = strongly agree) [72]. After completing the experiment, researchers collected demographic information of the participants (Cronbach’s α = 0.846). In the group of high cultural load, ancient style tea packaging box was taken as the stimulus material, in the group of low cultural load, an ordinary modern paper tea packaging box was taken as the stimulus material (See Fig 7A and 7B for details).

### 7.2 Experimental result

There was a main effect analysis. Experiment 4 conducted an analysis of variance (ANOVA) with purchasing intentions of tourist souvenirs as the dependent variable and traditional cultural load as the independent variable. The results showed that the purchasing intentions of tourist souvenirs in the high cultural load group (M = 5.69, SD = 1.358) was significantly higher than that in the low cultural load group (M = 2.71, SD = 1.37, F (1, 398) = 477.138, p<0.001). H1 was validated again.

**Fig 8:**
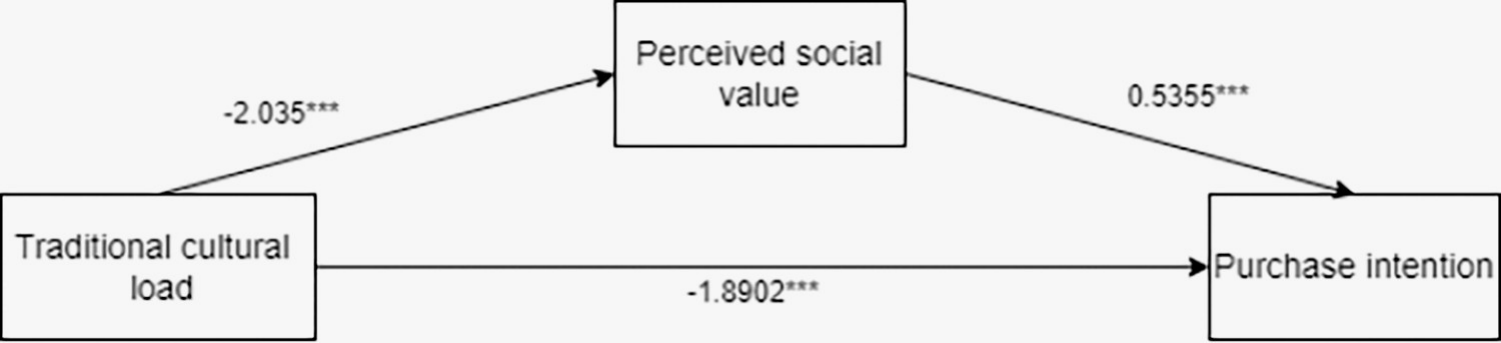
Path coefficient plot of mediating effects of perceived social value.

The researchers employed process model 4 to analyze the mediating role of perceived social value on the impact of traditional cultural load on tourists’ purchasing intentions of tourist souvenirs, by taking tourists’ purchasing intentions of tourist souvenirs as the dependent variable, traditional cultural load as the independent variable, and tourists’ perceived social value as the mediating variable (Bootstrap sample: 5000; [74]). The results showed that the mediating process of traditional cultural load—perceived social value—tourists’ purchasing intentions of tourist souvenirs was significant (β = -1.0898, SE = 0.1465, 95% CI [-1.3886 to -0.8080]). The coefficient of traditional cultural load—perceived social value is -2.035***, the coefficient of traditional cultural load—purchasing intentions is -1.8902***, and the coefficient of perceived social value—purchasing intentions is 0.5355***. It can be seen that perceived social value fully mediates the impact of traditional cultural load on tourists’ purchasing intentions of tourist souvenirs. H5 was validated (See Fig 8 for details).

### 7.3 Discussion

Experiment 4 verified the mediating role of perceived social value on the impact of traditional cultural load on tourists’ purchasing intentions of tourist souvenirs. The results showed that both the souvenir design with traditional cultural elements and the marketing strategies integrated with elements of social value play an important role in increasing tourists’ purchasing intentions. However, Experiment 4 failed to take into account the cultural heritage value of tourist souvenirs.

In order to compensate the shortcoming, Experiment 5 attempted to explore the mediating role of perceived heritage value on the impact of traditional cultural load on tourists’ purchasing intentions of tourist souvenirs.

## 8 Experiment 5: The mediating role of perceived value (perceived heritage value)

### 8.1 Experimental design

Experiment 5 took the one-way between-subjects design (traditional cultural load: high vs. low) in order to discuss the moderating role of perceived heritage value on the relationship between the traditional cultural load in tourist souvenirs and tourists’ purchasing intentions (H5), and to investigate the main effect of traditional cultural load in tourist souvenirs on tourists’ purchasing intentions (H1). There were 400 participants randomly recruited on Credamo, of which 200 (50%) were males and 200 (50%) were females. The age distribution of the participants was 2.8% (11) under the age of 18, 47.5% (190) aged 18–25, 42.5% (170) aged 26–40, 4% (16) aged 41–60, and 3.3% (13) aged 61 and above. They were randomly divided into two scenic spots of traditional tea town, one group of 200 participants with low traditional culture loaded products, and the other group of 200 participants with high traditional culture loaded tourist souvenirs.

**Fig 9:**
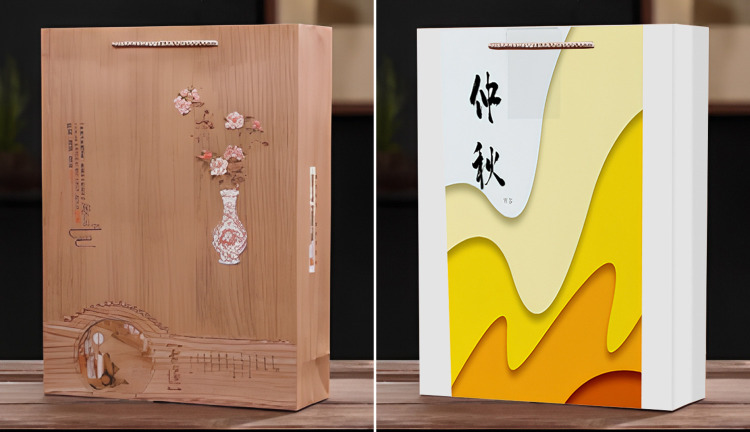
a. High traditional culture loading group stimulus material in experiment 5. b. Low traditional culture loading group stimulus material in experiment 5.

The materials for guidance in Experiment 5 were the same as in Experiment 4. The participants were led to answer the following questions, "How much do you think the traditional cultural load is in this product?", "Do you agree that the purchase of this product promotes its historical inheritance", "Do you agree that the purchase of this product extends the lifespan of the brand" (1 = Strongly Disagree, 7 = Strongly Agree) [77, 78], and "Do you agree that there is a great potential for you to purchase a cultural tourist souvenir from scenic spots after you learn about the cultural load in it"(1 = strongly disagree, 7 = strongly agree) [72]. After completing the experiment, researchers collected demographic information of the participants (Cronbach’s α = 0.719). Experiment 5 used the same stimulus material in Experiment 4 (See Fig 9A and 9B for details).

### 8.2 Experimental result

There was a main effect analysis. Experiment 5 conducted an analysis of variance (ANOVA) with purchasing intentions of tourist souvenirs as the dependent variable and the traditional cultural load as the independent variables. The results showed that the purchasing intentions of the high traditional cultural load group was significantly higher than that of the low traditional cultural load group (M _high cultural load_ = 5.7, SD _high cultural load_ = 1.576, M _low cultural load_ = 3.03, SD _low cultural load_ = 1.423, F (1, 397) = 316.215, P<0.001), and the traditional cultural load had a significant impact on the tourists’ purchasing intentions. H1 was again validated.

**Fig 10:**
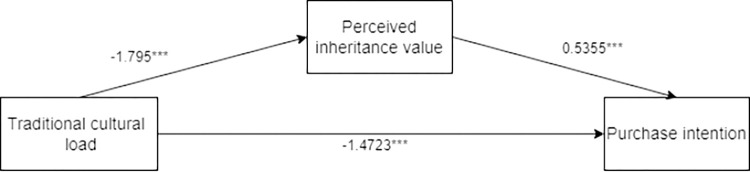
The mediating effect path coefficient of perceived heritage value.

There was an interaction effect analysis. The researchers employed process model 4 to analyze the mediating role of perceived heritage value on the impact of traditional cultural load on tourists’ purchasing intentions of tourist souvenirs, by taking traditional cultural load as the independent variable, tourists’ perceived heritage value as the mediating variable, and tourists’ purchasing intentions of tourist souvenirs as the dependent variable (Bootstrap sample: 5000; [74]). The results showed that the mediating process of traditional cultural load—perceived heritage values—tourists’ purchasing intention of tourist souvenirs was significant (β = -1.1977, SE = 0.1462, 95% CI [-1.4888 to -0.9157]). The coefficient of traditional cultural load—perceived heritage value is -1.795***, the coefficient of traditional cultural load—purchasing intentions is -1.4723***, and the coefficient of perceived heritage value—purchasing intentions is 0.6673***. It can be seen that the perceived heritage value fully mediates the impact of traditional culture on tourists’ purchasing intentions of tourist souvenirs (See Fig 10 for details).

### 8.3 Discussion

Experiment 5 verified the mediating role of perceived heritage value on the impact of traditional cultural load on tourists’ purchasing intentions of tourist souvenirs. The results showed that the perceived heritage value can effectively affect tourists’ purchasing intentions.

Experiment 5 failed to take into account tourists’ identity with traditional culture, and Experiment 6 attempted to explore the moderating role of tourists’ cultural identity on tourists’ purchasing intentions, and further analyze the mechanism by which traditional cultural load affects tourists’ purchasing intentions.

## 9 Experiment 6: The moderating role of cultural identity

### 9.1 Experimental design

Experiment 6 conducted a 2 (traditional cultural load: high vs. low) X 2 (cultural identity: high vs. low) ANOVA in order to discuss the moderating role of cultural identity on the relationship between the traditional cultural load in tourist souvenirs and tourists’ purchasing intentions, from the perspective of cultural attributes (H6), and to investigate the main effect of traditional cultural load in tourist souvenirs on tourists’ purchasing intentions (H1). There were 400 participants randomly recruited on Credamo, of which 200 (50%) were males and 200 (50%) were females. The age distribution of the participants was 4.8% (19) under the age of 18, 47.5% (190) aged 18–25, 42.5% (170) aged 26–40, 3.3% (13) aged 41–60, and 2% (8) aged 61 and above. They were randomly divided into two scenic spots of incense culture, one group of 200 participants with low traditional culture loaded products, and the other group of 200 participants with high traditional culture loaded tourist souvenirs.

**Fig 11:**
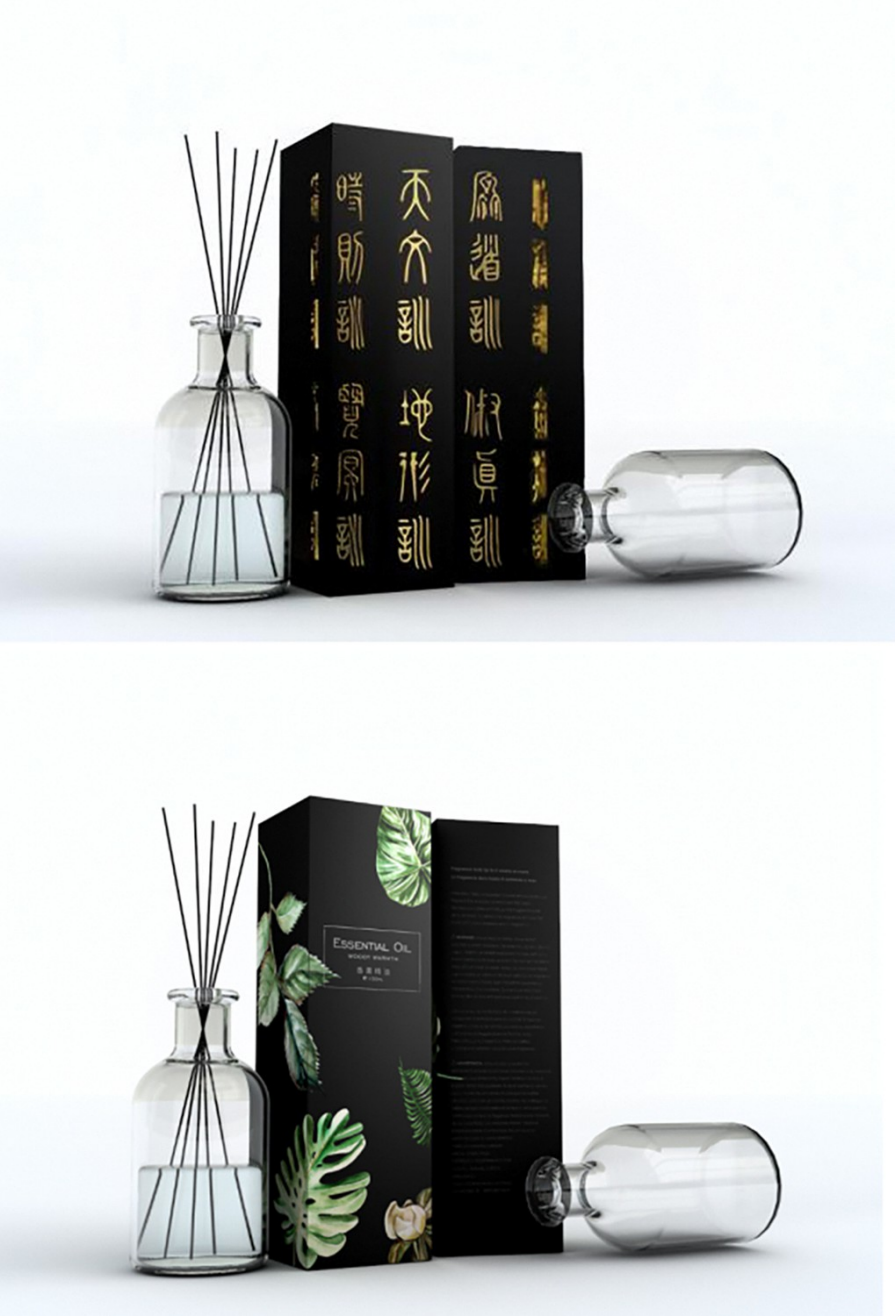
a. Experiment 6 of high traditional culture loading group stimulus materials. b. Experiment 6 of Low traditional culture loading group stimulus materials.

Participants were asked to imagine that they were relatively interested in incense culture and were in a large shopping mall in a scenic spot of incense culture, where are many local stores that make traditional incense, and a few modern incense-making stores. Then the participants were led to answer the following questions, "How much do you think the traditional cultural load is in this product?", together with three questions about cultural identity, "Do you agree that you are ready to take the time and learn about its history, traditions and customs", "Do you agree that you have a strong sense of belonging and attachment to your own ethnic group", and "Do you agree that you often do things to better understand your ethnic background" (1 = Strongly Disagree, 7 = Strongly Agree) [79], and "Do you agree that there is a great potential for you to purchase a cultural tourist souvenir from scenic spots after you learn about the cultural load in it" (1 = strongly disagree, 7 = strongly agree) [72]. After completing the experiment, researchers collected demographic information of the participants (Cronbach’s α = 0.737). In the group of high cultural load, incense decorated with oracle bone script was taken as the stimulus material, in the group of low cultural load, incense in an ordinary modern glass bottle was taken as the stimulus material (See Fig 11A and 11B for details).

### 9.2 Experimental result

There was a manipulation check of cultural identity. The results showed that M _Low Cultural Identity_ = 4, SD _Low Cultural Identity_ = 1.964; M _High Cultural Identity_ = 4.71, SD _High Cultural Identity_ = 1.822, F (1, 398) = 11.806, p<0.001. Therefore, the manipulations are effective in Experiment 6.

There was a main effect analysis. The researchers conducted an analysis of variance (ANOVA) with purchasing intentions of tourist souvenirs as the dependent variable and traditional cultural load as the independent variable. The results showed that the purchasing intentions of tourist souvenirs in the high cultural load group (M = 5.68, SD = 1.579) was significantly higher than that in the low cultural load group (M = 3.35, SD = 1.388, F (1, 398) = 245.794, p<0.001). H1 was again validated.

**Fig 12:**
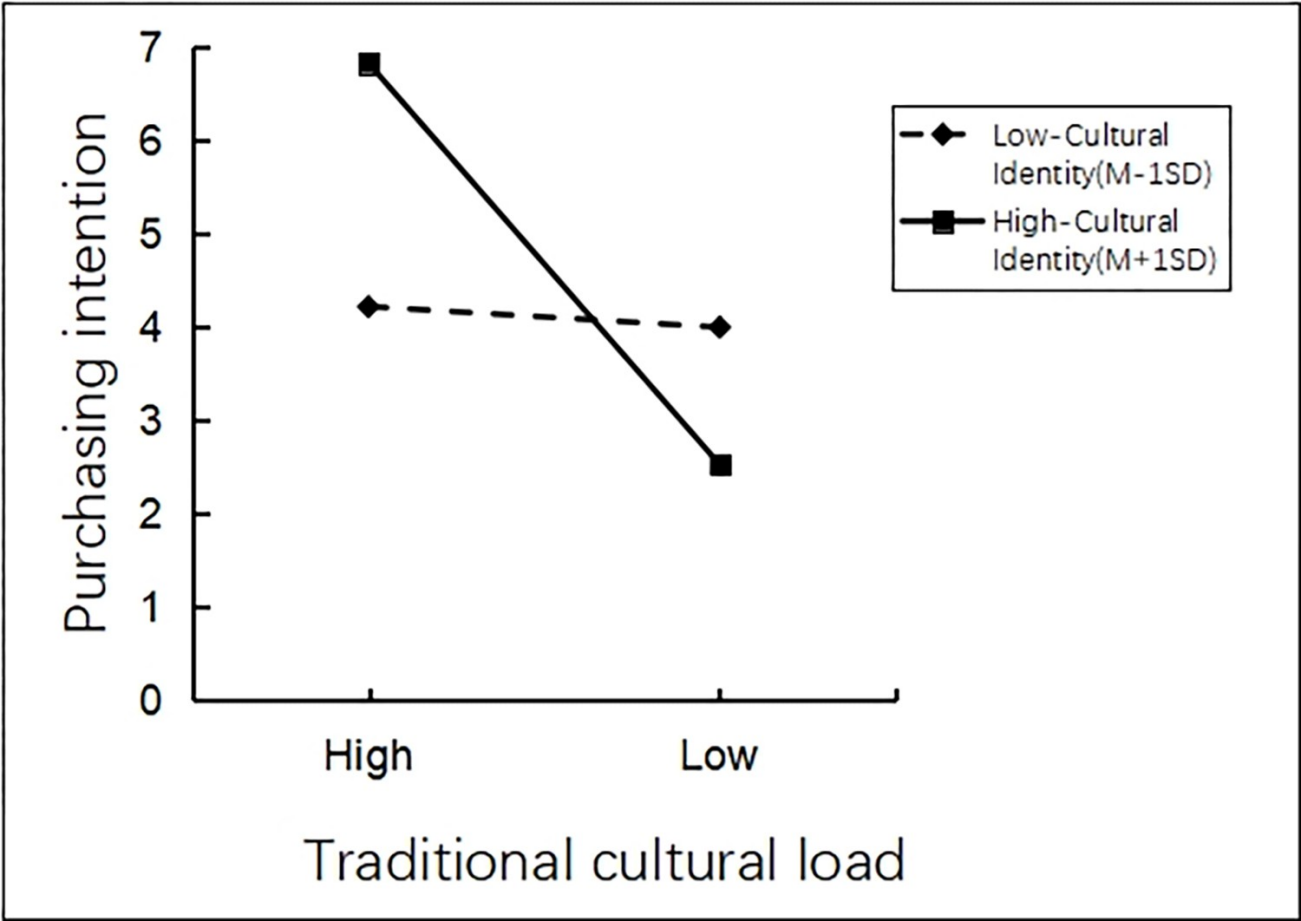
Interactive effect of cultural identity and traditional cultural load.

There was an interaction effect analysis. The researchers adopted Process Model 1 to analyze the moderating role in relationship between traditional cultural load and tourists’ purchasing intentions of tourist souvenirs, by taking traditional cultural load as the independent variable, cultural identity as the moderating variable, and tourists’ purchasing intentions as the dependent variable (Bootstrap sample: 5000; [74]). The results showed that the main effect of traditional cultural load on tourists’ purchasing intentions of tourist souvenirs in scenic spots was significant (β = -2.2659, P<0.001, 95%CI [-2.474~-2.0578]). The moderating effect of cultural identity on tourists’ purchasing intentions of tourist souvenirs was significant (β = 0.2057, P<0.001, 95%CI [0.1305~0.2808]). The interaction effect between cultural identity and traditional cultural load on tourists’ purchasing intentions of tourist souvenirs was significant (β = -1.4735, P<0.001, 95%CI [-1.6238~-1.3233]). It can be seen that the impact of traditional cultural load on tourists’ purchasing intentions of tourist souvenirs can be effectively moderated by cultural identity (See Fig 12 for details).

### 9.3 Discussion

Experiment 6 verified that cultural identity can effectively moderate the impact of traditional cultural load on tourists’ purchasing intentions of tourist souvenirs. The results showed that higher the cultural identity of tourists, the stronger their purchasing intentions of tourist souvenirs. The moderating effect of traditional cultural identity indicates that there is a potential impact of tourists’ own cultural attributes on their consumer behavior. These findings could bring some insights for tourism managers and marketers, who should pay more attention to promote souvenirs related to traditional culture, and provide corresponding products and services for tourists with high traditional cultural identity, to further increase their purchasing intentions.

## 10 Experiment 7: The moderating role of purchase purpose

### 10.1 Experimental design

Experiment 7 conducted a 2 (traditional cultural load: high vs. low) X 2 (purchase purpose: for oneself vs. for others) ANOVA in order to discuss the moderating role of purchase purpose on the relationship between the traditional cultural load in tourist souvenirs and tourists’ purchasing intentions (H7), and to investigate the main effect of traditional cultural load in tourist souvenirs on tourists’ purchasing intentions (H1). There were 804 participants randomly recruited on Credamo, 3 unfinished questionnaires have been excluded. A sample 801 participants was collected, of which 400 (49.9%) were males and 401 (50.1%) were females. The age distribution of the participants was 5.6% (45) under the age of 18, 40% (320) aged 18–25, 46.2% (370) aged 26–40, 3.2% (26) aged 41–60, and 5% (40) aged 61 and above. They were randomly divided into two scenic spots of incense museum, one group of 400 participants with low traditional culture loaded products, and the other group of 401 participants with high traditional culture loaded tourist souvenirs.

**Fig 13:**
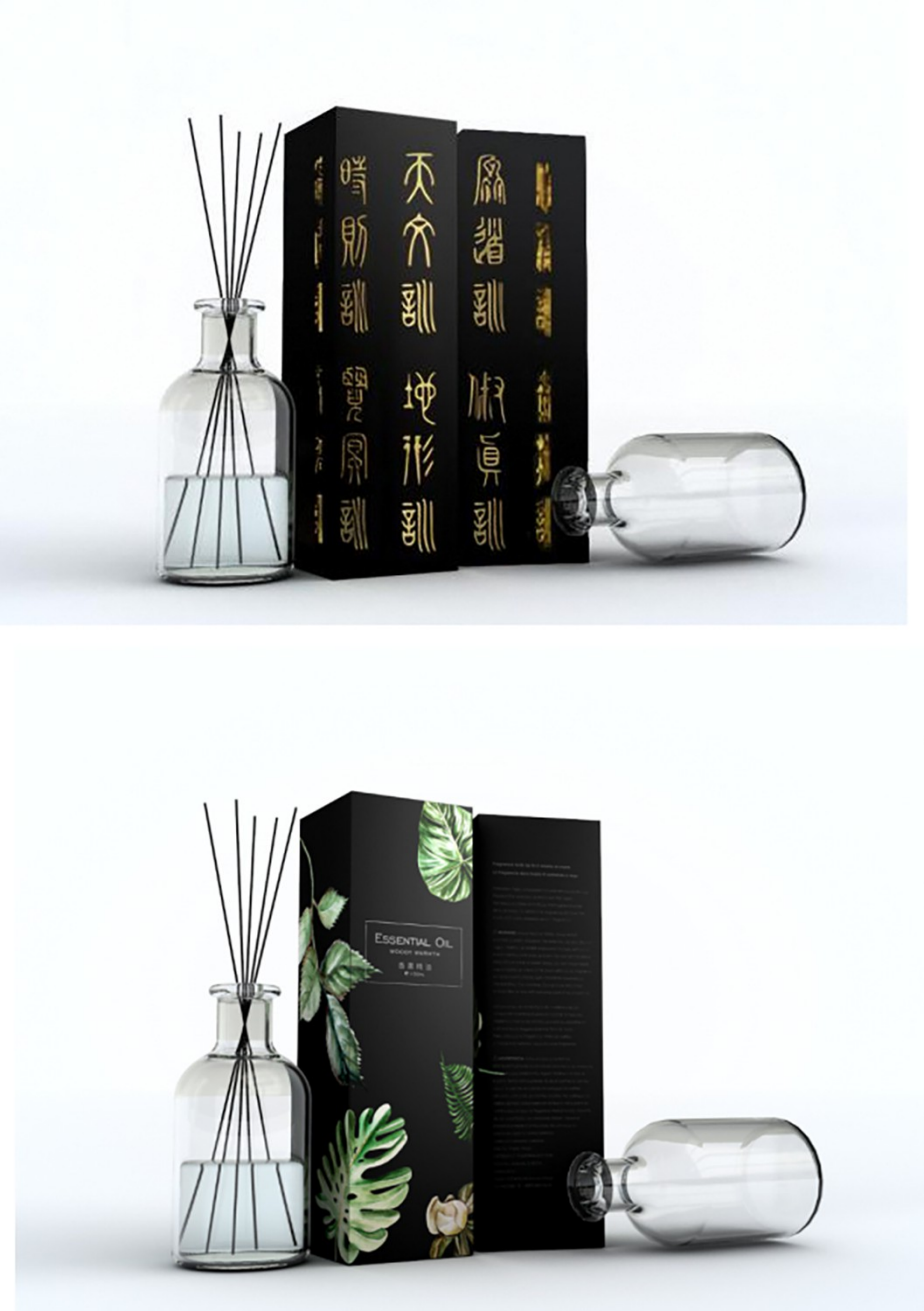
a. High traditional culture loading group stimulus material in experiment 7. b. Low traditional culture loading group stimulus material in experiment 7.

Participants were asked to imagine that they were intended to purchase incense and were in a cultural museum of incense, where sells incense with both modern design and traditional style, as well as has famous local masters of license production living nearby who carries excellent ancient license-making techniques. Then the participants were led to answer the following questions, "How much do you think the traditional cultural load is in this product?", "Do you agree that you are willing to purchase this tourist souvenir with traditional cultural load for yourself" (1 = Strongly disagree, 7 = Strongly agree), and "Do you agree that there is a great potential for you to purchase a cultural tourist souvenir from scenic spots after you learn about the cultural load in it" (1 = strongly disagree, 7 = strongly agree) [72]. After completing the experiment, researchers collected demographic information of the participants (Cronbach’s α = 0.784). The stimulus material used in experiment 7 was the same as in experiment 6 (See Fig 13A and 13B for details).

### 10.2 Experimental result

There was a manipulation check of purchase purpose. The results showed that M _purchase for oneself_ = 5.45, SD _purchase for oneself_ = 1.379, and M _purchase for others_ = 4.31, SD _purchase for others_ = 1.98, F (1, 799) = 88.862, p < 0.001. Therefore, the manipulations are effective in Experiment 7.

There was a main effect analysis. Experiment 7 conducted an analysis of variance (ANOVA) with purchasing intentions of tourist souvenirs as the dependent variable and traditional cultural load as the independent variables. The results about purchasing intentions showed that M _high cultural load_ = 5.78, SD _high cultural load_ = 1.346, M _low cultural load_ = 3.99, SD _low cultural load_ = 1.746, F (1, 799) = 351.122, p<0.001). The impact of traditional cultural load on tourists’ purchasing intentions in the high traditional cultural load group is significantly higher than in the low traditional cultural load group. H1 was validated again.

**Fig 14:**
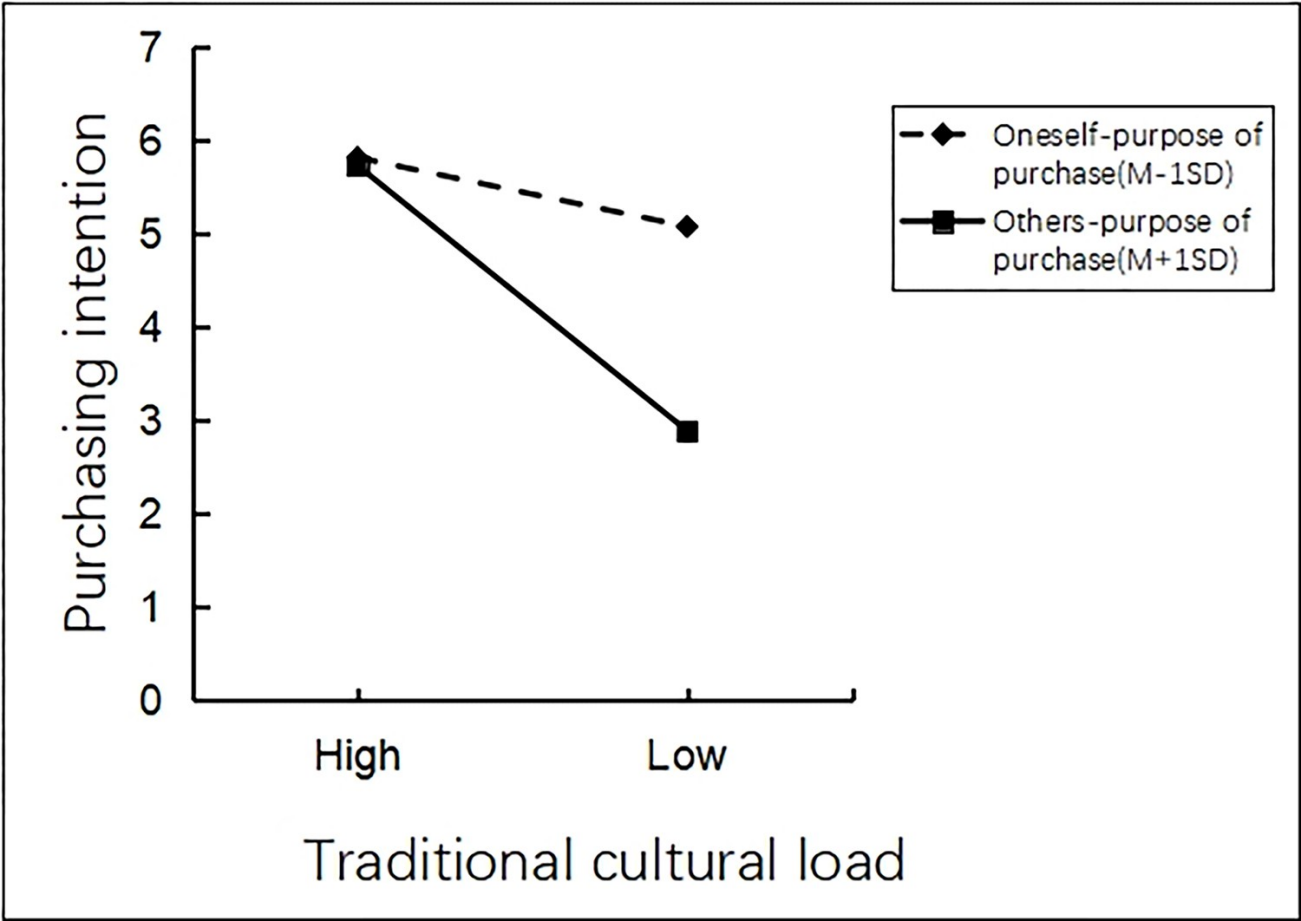
The interactive effect of purchase purpose and traditional cultural load.

There was an interaction effect analysis. The researchers employed process model 1 to analyze the moderating role of traditional cultural load on tourists’ purchasing intentions of tourist souvenirs, by taking traditional cultural load as the independent variable, purchasing purpose as the moderating variable and tourists’ purchasing intentions as the dependent variable, where high traditional cultural load = 1, low traditional cultural load = 2, purchasing for oneself = 1, and purchasing for others = 2 (Bootstrap sample: 5000; [74]). The results showed that the main effect of traditional cultural load on tourists’ purchasing intention of tourist souvenirs in scenic spots was significant (β = -1.7914, P<0.001,95% CI [-1.9792~-1.6036]). The moderating effect of purchase purpose on tourists’ purchasing intentions of tourist souvenirs was significant (β = -1.1386, P<0.001, 95%CI [-1.3264~-0.9508]). The interaction effect of purchase purpose and traditional cultural load on tourists’ purchasing intentions of tourist souvenirs was significant (β = -2.1046, P<0.001, 95%CI [-2.4802~-1.729]). It can be seen that purchase purpose effectively moderates the relationship between traditional cultural load and tourists’ purchasing intentions of tourist souvenirs (See Fig 14 for details).

### 10.3 Discussion

Experiment 7 further explored the moderating effect of traditional cultural load between the relationship of purchase purpose and tourists’ purchasing intentions. The results showed that the differences in tourists’ purchase purposes were relatively small in the context of high traditional cultural load, while the differences in tourists’ purchase purposes were larger in the context of low traditional cultural load. Tourists’ purchase purpose responds to the purchasing intentions to a certain extent, and traditional cultural load also promotes the tourists’ purchasing intentions. The interaction between purchase purpose and traditional cultural load can enhance tourists’ purchasing intentions of tourist souvenirs.

## 11 Discussion

During their travels, tourists make decisions about which souvenirs to purchase, and these choices reflect cultural values and preferences. Research consistently indicates that the cultural significance of souvenirs plays a pivotal role in tourists’ purchasing decisions [80], suggesting that the cultural load of souvenirs can impact the economic outcomes of the tourism industry. However, there is a gap in our understanding of how the traditional cultural load of souvenirs influences tourists’ psychological processes and decision-making. Thus, this paper addresses an underexplored issue within the field of tourism: Does the traditional cultural load of souvenirs influence tourists’ purchasing intentions?

We proposed and tested multiple hypotheses, and the validation of a series of hypotheses indicated that the traditional cultural load of souvenirs endows them with cultural significance, which in turn affects tourists’ assessments of the cultural value of souvenirs and subsequent purchasing intentions. Through experimentation, we confirmed that tourists indeed associate cultural significance with the characteristics of potential souvenirs and perceive these cultural meanings, consistent with the findings of Zhang et al. (2023), further validating that the cultural significance of souvenirs influences tourists’ consumption intentions. However, our study also further indicates that when the traditional cultural load of souvenirs is high, the purpose of purchase will further moderate tourists’ Purchasing Intention, which is not in line with the findings of Choo et al. (2023), who believe that ethnic identity does not affect Purchasing Intention. Our research found that tourists attach a sense of cultural confidence to products with high traditional cultural load in souvenirs, and they more strongly identify with their own ethnic culture. We further demonstrated that these deeply ingrained cultural meanings elucidate why the traditional craftsmanship, cultural representativeness, and innovation of souvenirs have cultural value and ultimately enhance the perceived cultural satisfaction of potential buyers.

We found that when the traditional cultural load is low, tourists with different hedonic purchase purposes exhibit varying Purchasing Intentions, with those having a higher hedonic purpose for souvenirs generating a higher Purchasing Intention. This is closely aligned with the research findings of Li et al. (2020), and our results also confirm that hedonic purposes indeed moderate individual consumer behavior among tourists. However, we observed a significant interaction effect between the practicality of souvenirs and tourists’ purchasing behavior. Reflecting on this interaction, it introduces a nuanced perspective to our understanding of the cultural impact of souvenirs. The interplay between the various characteristics of souvenirs and tourists’ purchasing purposes is a complex and variable mechanism; when tourists engage more deeply with the traditional cultural load embedded in souvenirs, they are more likely to assess the relationship between the souvenirs and traditional culture. At the same time, the purchasing purposes of tourists and the intrinsic practical value of the souvenirs themselves also effectively influence tourists’ Purchasing Intention.

According to Experiential Marketing Theory, consumer purchasing behavior is influenced not only by the functionality and price of a product but also by the experience during the purchase process. Experiential marketing emphasizes the creation and enhancement of interactive experiences between consumers and the brand or product, thereby increasing consumer satisfaction and loyalty. In the context of tourism souvenirs, this theory implies that enhancing the perceived value of souvenirs and the experience of the purchase process can effectively increase the buying intention of tourists. Therefore, we suggest enhancing the cultural experience of souvenirs, for example, by interactive exhibitions, storytelling, or cultural experience activities, allowing visitors to gain a deeper understanding of the culture and history behind the souvenirs. This enhances the perceived value of the souvenirs for tourists. Additionally, for tourists with different purposes for purchasing, souvenirs with strong local characteristics and storytelling can be designed to allow tourists to recall their journey through these items. The attractiveness of souvenirs can be increased by enhancing their cultural and historical content, such as providing introduction cards or digital content about the stories behind them. For tourists who purchase as gifts, they may focus more on the appearance and packaging of the souvenirs and whether they are suitable as gifts. Exquisite packaging services and customization options, such as personalized engraving or name printing, can be provided to increase the value of souvenirs as gifts.

## 12 Theoretical implications

First, the main contribution of this study is to introduce the unexplored concept of "traditional cultural load" to the field of tourism, even in marketing. Although previous studies have shown that traditional culture can affect tourists’ potential consumer behavior [81], few studies have examined the mechanisms by which the traditional cultural load in tourist souvenirs affects tourists’ purchasing intention, and the impact of the traditional cultural load in tourist souvenirs has not been valued. This study bridges this gap by investigating the impact of traditional cultural load (high vs. low) in tourist souvenirs on tourists’ purchasing intention. Cultural attributes of tourist souvenirs have been identified as important factors that affecting tourists’ purchasing intention [82], but the impact of the traditional cultural load (high vs. low) on tourists’ purchasing intention has not been thoroughly investigated. Echoing the recent call for research that focuses more on the cultural attributes of tourist souvenirs [1, 83, 84], this study advances the understanding of traditional culture and tourist souvenirs by including them in the research of tourism marketing. On the one hand, this study enriches the literature related to traditional culture and tourism marketing; on the other hand, the findings also extend the prior knowledge on the tourism marketing effects by emphasizing a new antecedent of tourists’ purchasing intention, which enables a better demonstration of how the traditional cultural load in tourist souvenirs (high vs. low) affects tourists’ purchasing intention.

Second, based on cultural identity theory, this study examines the moderating effect of cultural identity (high vs. low) on the traditional cultural load in tourist souvenirs (high vs. low) and tourists’ purchasing intention. Cultural identity is one of the important concepts in understanding the antecedents of consumers’ purchasing intention, and different degrees of consumers’ cultural identity may lead to different consumer behaviors [19]. Although cultural identity has gained comprehensive attention in marketing research, its effect in moderating the impact of traditional cultural load in tourist souvenirs on tourists’ purchasing intention remains unexplored. This study not only innovatively introduces the concept of traditional cultural identity of tourist souvenirs and widens the application of cultural identity in the tourist souvenirs marketing, but also explores the key role of cultural identity in moderating the relationship between traditional cultural load in tourist souvenirs on tourists’ purchasing intention. This study proves that tourists with higher traditional cultural identity develop their identification for tourist souvenirs with high traditional cultural load, thus increasing their purchasing intention. These findings enrich the knowledge about cultural identity theory and also contribute to better understand the influencing mechanism of traditional cultural load in tourist souvenirs affecting tourists’ purchasing intention.

Third, in the context of tourist souvenir marketing, based on the customer perceived value theory, this study examines the mediating role of customer perceived value on the relationship between traditional cultural load (high vs. low) in tourist souvenirs and tourists’ purchasing intention. Previous research has confirmed the impact of perceived value on consumer purchasing intention [85], however, how perceived value affects the relationship between traditional cultural load of tourist souvenirs and tourists’ purchasing intention has not been explored. This study advances the understanding by exploring the impact of traditional cultural load in tourist souvenirs on tourists’ purchasing intention. Although Srivastava et al. (2023) demonstrated that perceived value can effectively affect consumers’ purchasing intention, it remains unknown whether the perceived value theory can be applied to the researches of traditional cultural load in tourist souvenirs, as well as to positively enhance tourists’ purchasing intention. This study is one of the earlier attempts to validate the above issues based on customer perceived value theory. In addition, this study found that traditional culture has two dimensions of social value and heritage value. The perceived social value and perceived heritage value of the tourists for traditional cultural load in tourist souvenirs, are indirectly related to their personal attributes, which increases their purchasing intention in turn. Therefore, the results of this study not only expand the literature research of tourists’ consumer behavior, but also apply the customer perceived value theory to the marketing of traditional culture-enabled tourism products, while deepening the relevance of the customer perceived value theory to the study of traditional culture.

Last, this study also verifies the moderating role of product type (utilitarian vs. hedonic) and purchasing purpose (for oneself vs. for others) on the relationship between traditional cultural load (high vs. low) in tourist souvenirs and tourists’ purchasing intention. This study proves that tourists’ purchasing intention for tourist souvenirs with different traditional cultural load is not the same due to the differences in their utilitarian and hedonic nature. In addition, the difference in purchasing purposes (for oneself vs. for others) also significantly affects tourists’ purchasing intention. Accordingly, this study validates the moderating role of product type and purchasing purpose on the relationship between traditional cultural load (high vs. low) in tourist souvenirs and tourists’ purchasing intention.

## 13 Managerial implications

The findings of this study provide valuable inspirations for promoting traditional cultural empowerment in tourism development. First, it has significant implications for the application of traditional cultural elements in the design and marketing of tourist souvenirs. There are many cases of developing traditional culture-enabled tourism in contemporary tourism marketing, but the results have been disappointing. The results of the seven experiments conducted in this study consistently found that the inclusion of traditional cultural elements in the marketing of tourist souvenirs is sufficient to directly enhance tourists’ purchasing intention. Therefore, tourist souvenir designers and retailers should consider marketing from the perspective of high traditional cultural loads. Specifically, designers and retailers need to create souvenirs more highlight those traditional cultural characteristics. For example, tourist souvenirs should be designed based on the unique local traditional culture within scenic spots.

Second, this study found that product type, cultural identity, and purchasing purpose all have moderating effects on the relationship between traditional cultural load (high vs. low) in tourist souvenirs and tourists’ purchasing intention. Based on these findings, tourist souvenir retailers could focus on the following aspects to enhance tourists’ purchasing intention. Emphasis should be placed on the product type of tourist souvenirs. Studies have shown that product type can have an impact on tourists’ purchasing intention. Therefore, practitioners should carefully consider different types of tourist souvenirs and develop product strategies according to the needs of the target market [86]. For example, tourist with strong cultural identity need to be targeted to provide more souvenirs related to traditional culture. Emphasis should be placed on tourists’ cultural identity of tourist souvenirs. Studies have found that there is a moderating effect between tourists’ cultural identity and purchasing intention. Practitioners could highlight the cultural identity of the targeted consumer groups for tourist souvenirs. For example, incorporating local traditional elements into the design and marketing so as to attract tourists who have a strong identification with it. Emphasis should be placed on purchasing purpose of tourist souvenirs. The purchasing purpose of tourist souvenirs also has a moderating effect on purchasing intention. Therefore, practitioners can provide tourists with clearer purchasing purpose by highlighting the functions and usage of tourist souvenirs [87]. For instance, the theory of emotional design underscores the significance of leveraging emotions as a pivotal entry point to win consumers’ affection [88]. In the realm of souvenir marketing, it is essential to accentuate the value of these items as gifts, mementos, or collectibles to draw tourists who possess particular objectives for their purchases. There should be an increased focus on the emotional underpinnings of traditional culture within tourism souvenirs. According to research outcomes, augmenting the traditional cultural load can elevate the prospective buying intentions of tourists. We propose that industry practitioners should endeavor to incorporate a richer array of traditional cultural elements into souvenirs, encompassing aspects of design, materials, and patterns. In doing so, tourists will be better positioned to engage with and propagate the traditional culture of their travel destinations.

Third, this study found that perceived value mediates the relationship between traditional cultural load (high vs. low) in tourist souvenirs and tourists’ purchasing intention. Based on this finding, tourist souvenir designers and retailers could increase tourists’ purchasing intention with tourist souvenirs in line with the following two criteria. Specifically, only those tourist souvenirs that make tourists feel the heritage value and social value of the traditional culture can create an emotional connection between tourists and tourist souvenirs. Cultural identity theory emphasizes the sense of belonging and identification that individuals have towards their own cultural groups [89]. In the design of tourism souvenirs, incorporating local traditional cultural elements can stimulate tourists’ identification and interest in the destination’s culture, thereby enhancing the emotional connection between them and the souvenirs. This identification is not only about the recognition of cultural symbols but also a resonance with the deeper values and meanings behind the culture. Therefore, this study suggests that designers and retailers should first investigate whether the tourism souvenirs to be sold can provide tourists with sufficient traditional cultural perceived value and whether this perceived value can build an emotional connection between tourists and the souvenirs before designing and producing tourism souvenirs.

## 14 Limitations and future research

This study has two limitations. Firstly, due to the use of virtual scenarios, external validity may be limited [90]. Future research could improve external validity by conducting field studies. Secondly, while this study finds that tourist souvenirs with high traditional cultural load can increase purchasing intention and yield positive marketing outcomes, the long-term changes in purchasing intention remain unexplained. Cross-sectional studies cannot capture how purchasing intention changes over time and in different environments. Future studies could employ staged and cross-temporal data collection to further validate these findings. Moreover, subsequent research could explore other potential moderating variables, such as types of traditional culture and individual variation.

## Supporting information

S1 File Experimental data. (ZIP)

S2 File Appendix X longitudinal results. (DOCX)
