# Supplementary material for: How traditional cultural load affects tourists’ purchasing intention of tourist souvenirs
Source: PLoS One. 2025 Jan 9;20(1):e0313905. doi: 10.1371/journal.pone.0313905 (PMC11717242; doi:10.1371/journal.pone.0313905)
Supplement: S2 File — (DOCX) [file pone.0313905.s002.docx]

How does traditional cultural load affect tourists' purchase intention of tourist souvenirs

# 5 Experiment 1A: Traditional cultural payload -- purchase intention of tourist souvenirs

## 5.1 Experimental design

This study used a single-factor intersubject design experiment (high VS low traditional cultural load) to study the main effect of traditional cultural load of tourist souvenirs on tourists' purchase intention (H1). We divided the number of professional data collection platforms into two time periods (time period 1:August 25; Time slot 2:10, September) randomly convened 402 participants. Among them, 203 (50.5%) were male and 199 (49.5%) were female. The age groups of the participants were 47.3% (190 people) under the age of 18, 39.1% (157 people) between 18 and 25, 7% (28 people) between 26 and 40, and 6.7% (27 people) between 41 and 60. All subjects were randomly assigned to different scenarios: one scenario was a tourist souvenir with low traditional culture load (201 participants), and the other scenario was a tourist souvenir with high traditional culture load (201 participants).

Consistent with the cross-sectional experimental design, in the group of high cultural load, the tea set with Huainan sub-cultural elements was used as the stimulus material. In the low culture load group, we used ordinary tea sets without images as stimulus materials. We then instructed the subjects to imagine that they were shopping in a large shopping mall in a tea culture tourist area and were personally interested in traditional culture. The participants were asked "How much traditional culture do you think this product carries?" Then, the subjects were guided to answer "Do you agree that when you understand the cultural heritage of the product, you have a great possibility to buy cultural tourism souvenirs in the scenic spot (Fang,2012)?" Finally, we collected relevant demographic information of the subjects.

**5.2 Experimental Results**

Control check. We take purchase intention as the dependent variable and traditional culture load as the independent variable to conduct an independent sample T-test. The results showed that the subjects under the condition of high cultural load (M=6.41, SD=0.95) exhibited stronger tourist souvenirs than those under the condition of low cultural load (M=5.98, SD=1.567), t=3.349, d=1.296,P<0.001. It can be seen that experiment 1 was successfully manipulated.

Main effect analysis. We use purchase intention as the dependent variable and traditional cultural load as the independent variable to conduct one-way ANOVA. The results showed that the purchase intention (M high culture load =6.41, SD high culture load =0.95; M low culture load =5.98, SD low culture load =1.567), F (1,400) =11.217, P<0.001. The results show that the purchase intention of the subjects with high cultural load is significantly higher than that of the subjects with low cultural load. Therefore, H1 is validated.

## 5.3 Discussion

By collecting longitudinal experimental data, experiment 1A verified the main effect of traditional cultural loads in scenic spots on tourists' purchase intention of tourist souvenirs. This indicates that the traditional cultural load in the scenic spot will play a positive role in predicting the purchase intention of tourists. However, despite the above problems found in experiment 1A, experiment 1A failed to explain the internal mechanism and boundary conditions of traditional cultural load on the purchase intention of tourist souvenirs. In order to make up for the above shortcomings, we introduced product type (practical product: practical value) as a moderating variable in experiment 2A, and conducted longitudinal experiments to analyze the influence of the interaction between practical value and traditional cultural load on the purchase intention of tourist souvenirs.

**6 Experiment 2A: Moderating role of product type (****Utilitarian)**

## 6.1 Experimental design

In experiment 2, a single-factor inter-subject design (high vs. low traditional culture load) was used to explore the moderating effect of practical value on traditional culture load and tourists' purchase intention (H3), and the main effect of traditional culture load on tourists' purchase intention (H1) was investigated from the perspective of stimulus of product type (utilitarian product) (H2). We divided the number of professional data collection platforms into two time periods (time period 1:August 25; Time period 2:10 September) convene 379 subjects randomly. Among them, 189 (49.9%) were male and 190 (50.1%) were female. The age groups of the participants were 46.4%(176) under the age of 18, 42.7%(162) between 18 and 25, 5.3%(20) between 26 and 40, and 5.5%(21) between 41 and 60. The study subjects were randomly divided into two groups of tea culture tourism scenes, the first group was 189 people, the traditional culture load was low; Another group of 190 people had a higher traditional culture load.

The stimulus material was consistent with the design of cross-sectional experiment 2. We guided the subjects to imagine that they were shopping in a large shopping mall in a tea art culture tourist area, and they saw various videos of tea brewing techniques, books on the inheritance of tea art, and various tea products in different packages. The participants were then asked, "Do you agree that the tourist souvenirs with cultural loads meet your functional needs for such products?" (1 = strongly disagree, 7 = strongly agree) (Loiacono et al). Finally, the subjects were guided to answer the question of measuring the purchase intention of tourist souvenirs (Fang,2012). Finally, we calculated demographic information (Cronbach's α =0.762). Booklike tea box packaging was used in the high-culture load group; In the low culture load group, ordinary tin tea packaging was used, which was consistent with the original cross-sectional experimental material.

## 6.2 Experimental Results

Main effect test. We take the purchase intention of tourist souvenirs as the dependent variable and the traditional cultural load as the independent variable to conduct ANOVA. The results showed that the purchase intention of tourist souvenirs in the high culture load group (M=6.56, SD=0.708) was significantly higher than that in the low culture load group (M=6.07, SD=1.446, F (1,377) =17.52, P<0.001). H1 is verified.

Analysis of regulatory effects. We took the traditional culture load as the independent variable, the product practical value as the moderating variable, and the purchase intention of tourist souvenirs as the dependent variable. process model 1 was adopted to analyze the regulatory relationship between the product practical value and the traditional culture load and the purchase intention of tourist souvenirs (Bootstrap sample: 5000; Igartua & Hayes, 2021). The results showed that the main effect of traditional culture load on the purchase intention of tourist souvenirs was significant (β=0.4738, P<0.001,95%CI[0.2578~0.6899]). The significant effect of product practical value on purchasing intention of tourist souvenirs (β=0.2598, P<0.001,95%CI[0.1833~0.3363]). The interaction of product practical value and traditional cultural load on purchasing intention of tourist souvenirs (β=-0.1694, P<0.001,95%CI[-0.3224, -0.0164]). Therefore, the practical value of products can effectively regulate the traditional cultural load and the purchase intention of tourist souvenirs, and verify hypothesis H3. The diagram of specific interaction effects is shown in Figure 1.

Figure 1.

## 6.3 Discussion

Experiment 2A verifies that the practical value of products can effectively regulate the influence of traditional cultural load on the purchase intention of tourist souvenirs through longitudinal experimental data. When considering the practical attributes of products, tourists are more inclined to buy tourist souvenirs with higher practical value and higher traditional cultural load. When the cultural load of products is the same, tourists tend to buy products with higher practical value. Despite these findings, experiment 2A did not examine hedonic properties in product types. In order to make up for the shortcomings of the above experiments, this study introduced the hedonic element of product type as a moderating variable in experiment 3A to analyze the moderating effect of traditional cultural loads on the purchase intention of tourist souvenirs.

**7 Experiment 3A: Moderating role of product type (****Hedonic)**

7.1 Experimental design

In experiment 3A, ANOVA of 2(traditional cultural load: high vs low)X 2(product hedonic attribute: high vs low) was conducted to explore the regulating effect of hedonic products in scenic areas on traditional cultural load of tourist souvenirs and tourists' purchase intention from the perspective of stimulus of product type (hedonic product) (H4). The main effect of traditional cultural load of tourist souvenirs on tourists' purchase intention was investigated (H1). We divided the number of professional data collection platforms into two time periods (time period 1:August 25; Time slot 2:10, September) convened a random group of 380 participants. There were 186 males (48.9%) and 194 females (51.1%). 46.1%(175) were under the age of 18, 40.8%(155) were between 18 and 25, 7.6%(29) were between 26 and 40, and 5.5%(21) were between 41 and 60. The study subjects were randomly divided into two groups of tea culture tourism scenarios: low culture load group (192 participants); High literacy group (188 participants).

Specifically, we used the same guided materials in experiment 3A as in experiment 2A. The participants were then asked, "Do you agree that the cultural souvenirs mentioned above can bring you pleasure and make you feel happy during the shopping process?" (1 = strongly disagree, 7 = strongly agree) (Loiacono et al). Finally, participants were guided to answer questions measuring souvenir purchase intention (1 = strongly disagree, 7 = strongly agree) (Fang,2012). Finally, we counted demographic information (Cronbach's α =0.73). Booklike tea box packaging was used in the high-culture load group; In the low culture load group, ordinary tin tea packaging was used, which was consistent with the original cross-sectional experimental material.

## 7.2 Experimental Results

Main effect test. We take the purchase intention of tourist souvenirs as the dependent variable and the traditional cultural load as the independent variable to conduct ANOVA. The data show that the purchase intention (M high cultural load =6.46, SD high cultural load =0.95, M low cultural load =6.07, SD low cultural load =1.577), F (1,378) =8.477, P<0.001), the data result shows that the high traditional cultural load is significantly higher than the low traditional cultural load. And the traditional cultural load has a significant impact on the purchase intention of tourists. H1 is verified.

Analysis of regulatory effects. With traditional cultural load as the independent variable, product hedonic value as the moderating variable, and purchase intention of tourist souvenirs as the dependent variable, we analyzed the moderating effect of product hedonic value using process model 1 (Bootstrap sample: 5000; Igartua & Hayes, 2021). The results showed that the main effect of traditional culture load on the purchase intention of tourist souvenirs was significant (β=0.3471, P=0.005,95%CI[0.1052~0.5891]). The effect of product hedonic value on the purchase intention of tourist souvenirs was significant (β=0.2933, P<0.001,95%CI[0.2221~0.3645]). The interaction between product hedonic value and traditional cultural load on tourists' purchase intention of tourist souvenirs was significant (β=-0.1554, P=0.032,95%CI[-0.2979~-0.0129]). Therefore, the hedonic value of products can effectively regulate the traditional cultural load and the purchase intention of tourists for tourist souvenirs, and verify hypothesis H4. The concrete interaction effect diagram is shown in Figure 2.

Figure 2.

## 7.3 Discussion

Experiment 3A explains the moderating effect of hedonic value on the traditional cultural load on the purchase intention of tourist souvenirs through longitudinal experimental analysis. The results show that under the background of high cultural load, tourists are more inclined to buy tourist souvenirs with high hedonic value, while under the background of low cultural load, tourists are more inclined to buy tourist souvenirs with low hedonic value. However, experiment 3A did not consider the influence of the perceived value of tourist souvenirs on tourists' purchase intention. Consumers' perception of cultural inheritance factors and social value factors of tourist souvenirs may further affect tourists' purchase intention of tourist souvenirs. Therefore, in experiment 4, we studied the mediating effect of tourists' perceived social value on traditional cultural loads on tourists' tourism souvenirs.

**8 Experiment 4A: The mediating role of perceived value (perceived social value)**

## 8.1 Experimental design

Experiment 4A adopted a single-factor intersubject design (high vs low traditional cultural load) to explore the mediating role of perceived social value in the relationship between traditional cultural load of tourist souvenirs and tourists' purchase intention (H4). We divided the number of professional data collection platforms into two time periods (time period 1:August 25; Session 2: September 10) convened a random group of 368 participants. Among them, 185 (50.3%) were male and 182 (49.5%) were female. The population was spread out as 45.9%(169) under the age of 18, 42.4%(156) from 18 to 25, 7.1%(26) from 26 to 40, and 4.6%(17) from 41 to 60. The study subjects were randomly divided into two groups of travel scenarios: low culture load group (186 participants); High culture load group (182 participants).

The stimulus material was consistent with the design of cross-sectional experiment 4A. In experiment 4A, we guided the subjects to imagine that they were traveling in the traditional tea town tourist attraction, where there were many products with antique elements and modern elements interwoven. When they were browsing the products in the large shopping mall of the tourist attraction, the subjects would be asked: "What do you think is the traditional cultural load of the product?" . Participants were then asked about measures of perceived value, such as "Do you agree or disagree that the aforementioned cultural memorials help you to be recognized by others" (1 = strongly disagree, 7 = strongly agree) (Sweene & Soutar.2001). Then, the subjects were guided to answer the measurement questions requiring the purchase intention of tourist souvenirs (Fang,2012). Finally, demographic information was collected (Cronbach's α =0.846). The high culture load group took ancient tea packaging box as the stimulus material, and the low culture load group took ordinary modern paper tea packaging box as the stimulus material.

## 8.2 Experimental Results

Main effect test. We take the purchase intention of tourist souvenirs as the dependent variable and the traditional cultural load as the independent variable to conduct ANOVA. The results showed that the purchase intention of tourists in the high culture load group (M=6.37, SD=0.753) was significantly higher than that in the low culture load group (M=6.01, SD=1.346, F (1,366) =10.434, P<0.001). H1 is verified.

Mediating effect test. Taking purchasing intention of tourist souvenirs as the dependent variable, traditional cultural load as the independent variable, and tourists' perceived social value as the mediating variable, we analyzed the mediating effect of perceived social value by using process model 4 (Bootstrap sample: 5000; Igartua&Hayes, 2021). The results showed that the mediating process of traditional cultural loads-perceived social values-purchase intention of tourists was significant (β=0.1129, SE=0.0452, 95%CI[0.0319~0.206]). Among them, the coefficient of traditional cultural load - perceived social value is 0.2955**, the coefficient of traditional cultural load - purchase intention is 0.2554*, and the coefficient of perceived social value - purchase intention is 0.382***. Therefore, the perceived social value is a complete intermediary between the traditional culture and the purchase intention of tourist souvenirs. Verify hypothesis H5. The resulting mediation effect chain diagram is shown in Figure 3.


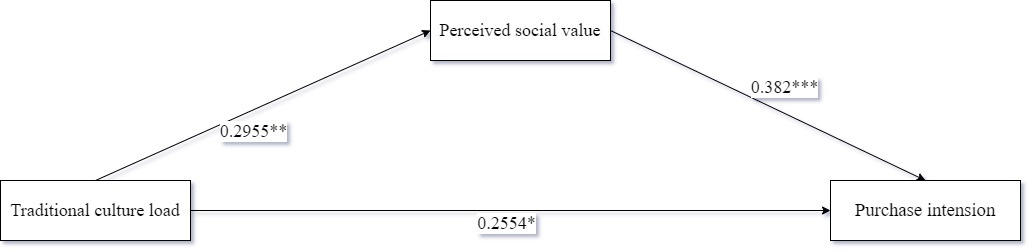


Figure 3.

## 8.3 Discussion

Experiment 4A verified the effect of perceived social value mediating traditional cultural load on tourists' purchase intention of tourist souvenirs and the main effect of traditional cultural load and purchase intention. The results show that when tourists perceive the high social value of traditional cultural souvenirs, their willingness to buy such souvenirs will increase. Integrating social value elements into souvenir design and marketing strategy with traditional cultural elements plays an important role in improving tourists' purchase intention. But when the traditional cultural elements are given to the product, the product also has the potential cultural inheritance value. In experiment 5, we tried to explore whether perceived heritage value would mediate the relationship between traditional cultural load and tourist souvenir purchase intention.

**9 Experiment 5A: The mediating role of perceived value (****perceived heritage value)**

## 9.1 Experimental design

Experiment 5A adopted a single-factor design (high vs. low traditional cultural load) to explore the moderating effect of perceived heritage value on the relationship between traditional cultural load of tourist souvenirs and tourists' purchase intention (H3). We divided the number of professional data collection platforms into two time periods (time period 1:August 25; Session 2: September 10) convened 382 participants at random. Among them, 189 (49.5 percent) were male and 193 (50.5 percent) were female. There were 175 people (45.8%) under the age of 18, 149 (39%) from 18 to 25, 24 (6.3%) from 26 to 40, and 34 (8.9%) from 41 to 60. The study subjects were randomly divided into two groups of travel scenarios: low cultural load group (197 participants); High culture load group (185 participants).

We used the same guiding and stimulating materials in experiment 5A as in experiment 4. The participants were asked, "When you see this product, what do you think is the traditional cultural payload of this product?" . Subsequently, participants were asked to measure the perceived heritage value, such as "buying this product can promote the historical heritage of the product" (1 = strongly disagree, 7 = strongly agree) (Morhart et al,2015; Napoli et al,2014). Finally, the subjects were guided to answer the measurement questions of purchasing intention of tourist souvenirs (1 = strongly disagree, 7 = strongly agree) (Fang,2012). Finally, we collected demographic information.

## 9.2 Experimental Results

Main effect test. We take the purchase intention of tourist souvenirs as the dependent variable and the level of traditional cultural load as the independent variable to conduct ANOVA. The research results show that the purchase intention (M high culture load =6.44, SD high culture load =1.042, M low culture load =5.82, SD low culture load =1.698), F (1,380) =18.569, P<0.001), the data results show that, Compared with those with low cultural load, tourists show a higher willingness to buy souvenirs with high traditional cultural load. H1 is verified.

Mediation analysis. Taking traditional culture load as the independent variable, perceived inheritance value as the mediating variable, and purchase intention of tourist souvenirs as the dependent variable, we adopted process model 4 to analyze the mediating effect of perceived inheritance value on traditional culture load and tourists' purchase intention of tourist souvenirs (Bootstrap sample: 5000; Igartua&Hayes, 2021). The results showed that the mediating process of traditional cultural load -- perceived heritage value -- purchase intention of tourist souvenirs was significant (β=-0.1685, SE=0.0751, 95%CI[-0.3207~-0.0261]). Among them, the coefficient of traditional cultural load - perceived heritage value is -0.308*, the coefficient of traditional cultural load - purchase intention is -0.4575***, and the coefficient of perceived heritage value - purchase intention is 0.5469***. Therefore, the perceived inheritance value is a complete intermediary between traditional culture and the purchase intention of tourist souvenirs. Verify hypothesis H5. The chain intermediary path coefficient of perceived inheritance value is shown in Figure 4.


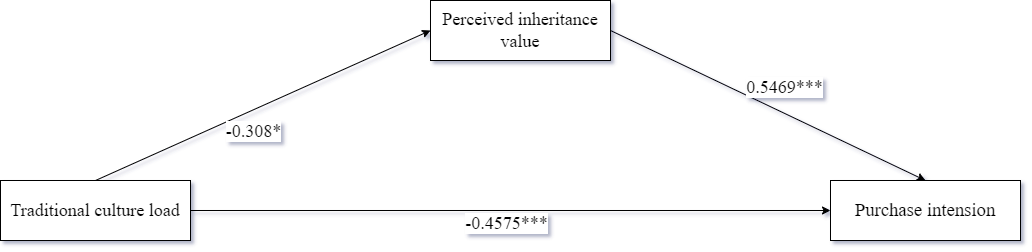


Figure 4.

## 9.3 Discussion

Experiment 5A verified the mediating effect of perceived heritage value on traditional culture load and tourists' purchase intention of tourist souvenirs. The results show that the perceived heritage value of products can effectively affect the purchase intention of tourists. The higher the perceived heritage value of tourist souvenirs, the stronger the purchase intention of tourists. At the same time, the traditional cultural load can also directly affect the purchase intention of tourists. The higher the traditional cultural load, the stronger the purchase intention of tourists. Despite the above findings in experiment 5, Experiment 5 did not take into account the influence of cultural attributes of works on tourists' purchase intention. Therefore, in experiment 6, we explored whether tourists' cultural identity can effectively regulate tourists' purchase intention, and further analyzed the internal mechanism of traditional cultural load on tourists' purchase intention.

**10 Experiment 6A: The moderating role of cultural identity**

## 10.1 Experimental design

The purpose of experiment 6A is to verify the moderating effect of cultural identity on the traditional cultural load of souvenirs and tourists' purchase intention, and the main effect of traditional cultural load on tourists' purchase intention of tourist souvenirs (H1). We designed a 2 (traditional cultural load: high VS low) X2 (cultural identity: high VS low) variance experiment. We divided the number of professional data collection platforms into two time periods (time period 1:August 25; Session 2: September 10) convened a random group of 406 participants. Among them, 208 (51.2%) were male and 198 (48.8%) were female. The age ratio of the subjects was 24.4% (99) under the age of 18, 53.2% (216) between 18 and 25, 16.5% (67) between 26 and 40, 4.7% (19) between 41 and 60, and 1.2% (5) aged 61 and above. We randomly divided the study subjects into two groups of travel scenarios: low cultural load group (200 participants); High culture load group (206 participants).

The stimulus material was consistent with the design of cross-sectional experiment 4. We asked the participants to imagine that they were traveling in a scenic area of incense culture, where there were many shops making traditional incense and modern incense making shops. While browsing the product in a large shopping mall in a tourist area, the participants were asked: What do you think is the traditional cultural payload of the product? Subsequently, participants were asked questions about cultural identity, such as "Do you agree that you are willing to spend time and learn about its history, traditions and customs" (1 = strongly disagree, 7 = strongly agree) (Phinney.2007). Finally, the subjects were guided to answer the question of purchasing intention of tourist souvenirs in scenic spots (1 = strongly disagree, 7 = strongly agree) (Mathwick et al, 2001). Finally, we counted demographic information (Cronbach's α =0.737). In the high-culture load group, an oracle-shaped aromatherapy was used; Common modern glass bottle aromatherapy was used in the low culture load group.

## 10.2 Experimental Results

Main effect test. We take the purchase intention of tourist souvenirs as the dependent variable and the traditional cultural load as the independent variable to conduct ANOVA. The results showed that the purchase intention of tourists in the high culture load group (M=6.1, SD=1.416) was significantly higher than that in the low culture load group (M=5.68, SD=1.579, F (1,404) =, P<0.001). H1 is verified.

Analysis of regulatory effects. We take traditional cultural load as the independent variable, cultural identity as the moderating variable, and purchase intention of tourist souvenirs as the dependent variable. process model 1 was used to analyze the moderating effect of cultural identity (Bootstrap sample: 5000; Igartua & Hayes, 2021). The results showed that the main effect analysis of traditional culture load on tourists' purchase intention of tourist souvenirs was significant (β=0.3963, P<0.001,95%CI[0.1817-0.615]). The significant effect of cultural identity on the purchase intention of tourist souvenirs (β=0.7041, P<0.001,95%CI[0.6229~0.7852]). The interaction between cultural identity and traditional cultural load on tourist souvenir purchase intention was significant (β=-0.4698, P<0.001,95%CI[-0.6319~-0.3077]). Therefore, cultural identity has a significant moderating effect on traditional cultural load and purchase intention of tourist souvenirs. The result of the interaction effect path between cultural identity and traditional cultural load is shown in Figure 5.

Figure 5.

## 10.3 Discussion

Experiment 6A verified that cultural identity can effectively regulate the effect of traditional cultural loads on tourists' purchase intention of tourist souvenirs. Under the background of high cultural load, the higher the cultural identity of tourists, the stronger the purchase intention; In the context of low cultural load, the higher the cultural identity of tourists, the lower the purchase intention. However, from the perspective of tourists' purchase purpose, there is still a large research gap to explore whether tourists' purchase of tourist souvenirs will be affected by the purchase purpose. Therefore, in experiment 7, from the perspective of purchase purpose, we analyzed the differences between tourist souvenirs purchased for others and purchased for themselves and the differences in purchase intention under different cultural load backgrounds.

**11 Experiment 7A: The moderating role of purchase purpose**

## 11.1 Experimental design

The purpose of experiment 7A is to explore the moderating effect of tourists' sense of purchase purpose on the purchase intention of souvenirs with traditional cultural loads from the perspective of tourists' purchase purpose. We designed 2 (traditional culture load: high VS low) X2 (purchase purpose: self VS others) variance experiment, which was divided into two time periods on the professional data collection platform (time period 1:August 25; Time slot 2:10 September) convened 733 participants randomly. Among them, 357 (48.7%) were male and 376 (51.3%) were female. The age ratio of the subjects was 47.5% (348) under the age of 18, 40.7% (298) between 18 and 25, 5.2% (38) between 26 and 40, and 6.7% (49) between 41 and 60. The study subjects were randomly divided into two groups of travel scenarios: low culture load group (343 participants); High culture load group (390 participants).

We used the same guiding and stimulating materials in experiment 7A as in experiment 6A. Subsequently, the subjects were asked "Do you agree that you are willing to buy the above tourist souvenirs containing traditional cultural loads for friends or family members", and the question asked for themselves was "Do you agree that you are willing to buy the tourist souvenirs containing traditional cultural loads for yourself" (1 = strongly disagree, 7 = strongly agree). Finally, the subjects were guided to answer the question of purchasing intention of tourist souvenirs in scenic spots (1 = strongly disagree, 7 = strongly agree) (Mathwick et al, 2001). Finally, we collected demographic information.

## 11.2 Experimental Results

Main effect test. We take the purchase intention of tourist souvenirs as the dependent variable and the traditional cultural load as the independent variable to conduct ANOVA. The data show that the purchase intention (M high cultural load =6.38, SD high cultural load =1.176, M low cultural load =5.95, SD low cultural load =1.334), F (1,731) =21.429, P<0.001). The data result shows that the high traditional cultural load is significantly higher than the low traditional cultural load. Traditional cultural load has a significant impact on tourists' purchase intention. H1 is verified.

Interaction effect analysis. We take the traditional cultural load as the independent variable, the purchase purpose as the moderating variable, and the purchase intention of tourists as the dependent variable. We used process model 1 to analyze the moderating effect of purchase purpose (Bootstrap sample: 5000; Igartua & Hayes, 2021). The results showed that the main effect of traditional culture load on tourists' purchase intention of tourist souvenirs was significant (β=0.4533, P<0.001,95%CI[0.2788-0.6277]). Purchase purpose significantly affected the purchase intention of tourists (β=0.7052, P<0.001,95%CI[0.5311~0.8793]). Purchase purpose and traditional cultural load had significant interaction on tourists' purchase intention of tourist souvenirs (β=-0.3558, P=0.045,95%CI[-0.7047~-0.0069]). Therefore, the purchase purpose can effectively regulate the relationship between the traditional cultural load and the purchase intention of tourists. The interactive effect of purchase purpose and traditional cultural load is shown in Figure 6.

Figure 6.

## 11.3 Discussion

Experiment 7A further explained the moderating effects of traditional cultural load and purchase purpose on tourists' purchase intention. According to the experimental results, no matter under any cultural load background, the purchase intention of tourists to buy tourism souvenirs for themselves is higher than that of others. However, under the background of high traditional cultural load, the difference of tourists' purchase purpose is relatively small, while under the premise of low traditional cultural load, the difference of tourists' purchase purpose is larger. The purchase purpose reflects the purchase motive of tourists to a certain extent, and the conditions of traditional cultural load also stimulate and promote the purchase motive of tourists. The interaction between purchase purpose and traditional cultural load can enhance tourists' purchase intention and make them more willing to buy tourist souvenirs in scenic spots.
